# Developing comprehensive woman hand-held case notes to improve quality of antenatal care in low-income settings: participatory approach with maternal health stakeholders in Malawi

**DOI:** 10.1186/s12913-024-10922-3

**Published:** 2024-05-15

**Authors:** Leonard Mndala, Chifundo Kondoni, Luis Gadama, Catherine Bamuya, Annie Kuyere, Bertha Maseko, Fannie Kachale, Mtisunge Joshua Gondwe, David Lissauer, Alinane Linda Nyondo-Mipando

**Affiliations:** 1 Malawi-Liverpool-Wellcome Programme, Blantyre, Malawi; 2 University of Liverpool, Liverpool, UK; 3 Kamuzu University of Health Sciences, Blantyre, Malawi; 4 Malawi Epidemiological and Intervention Research Unit, Karonga, Malawi; 5 Ministry of Health, Lilongwe, Malawi

**Keywords:** Antenatal care, Woman hand-held case notes, Health passport book, Healthcare delivery, Pregnancy experience, Pregnancy, Low-income countries, Quality improvement, Malawi

## Abstract

**Background:**

In the quest for quality antenatal care (ANC) and positive pregnancy experience, the value of comprehensive woman hand-held case notes cannot be emphasised enough. However, the woman’s health passport book in Malawi presents gaps which hinder provision of quality care, especially during pregnancy. We aimed to develop a compressive updated woman hand-held case notes tool (health passport book) which reflects WHO 2016 ANC guidelines in Malawi.

**Methods:**

From July 2022 to August 2022, we applied a co-creative participatory approach in 3 workshops with key stakeholders to compare the current ANC tool contents to the WHO 2016 ANC guidelines, decide on key elements to be changed to improve adherence and change in practice, and redesign the woman’s health passport tool to reflect the changes. Within-group discussions led to whole-group discussions and consensus, guided by a modified nominal group technique. Facilitators guided the discussions while ensuring autonomy of the group members in their deliberations. Discussions were recorded and transcribed. Data was analysed through thematic analysis, and reduction and summaries in affinity diagrams. The developed tool was endorsed for implementation within Malawi’s healthcare system by the national safe motherhood technical working group (TWG) in July 2023.

**Results:**

Five themes were identified in the analysis. These were (i) critical components in the current tool missed, (ii) reimagining the current ANC tool, (iii) opportunity for ultrasound scanning conduct and documentation, (iv) anticipated barriers related to implementation of the newly developed tool and (v) cultivating successful implementation. Participants further recommended strengthening of already existing policies and investments in health, strengthening public private partnerships, and continued capacity building of healthcare providers to ensure that their skill sets are up to date.

**Conclusion:**

Achieving goals of quality ANC and universality of healthcare are possible if tools in practice reflect the guidelines set out. Our efforts reflect a pioneering attempt in Malawi to improve women’s hand-held case notes, which we know help in enhancing quality of care and improve overall women’s satisfaction with their healthcare system.

**Supplementary Information:**

The online version contains supplementary material available at 10.1186/s12913-024-10922-3.

## Background

The World Health Organisation (WHO) has recommended that every pregnant woman carries her case notes during pregnancy to improve continuity, quality of care, and overall pregnancy experience [1]. Evidence from a comprehensive review indicated that the benefits of pregnant women carrying case notes outweigh the disadvantages; with many women reporting an improvement in their sense of control over their health and utilisation of maternal health services [2]. Furthermore, the use of an improved maternal and child health card increased antenatal care visits in Mongolia [3].

Generally, pregnant women want a positive pregnancy experience characterised by a sense of control and empowerment over their health, support, and trust in their healthcare system and continuity of care throughout pregnancy [4]. Women’s hand-held case notes have the potential to bring a sense of positive pregnancy experience if they are comprehensive, educative, private, and offer a sense of empowerment. From the healthcare provider’s perspective, wide-ranging case notes in antenatal care services improve the availability of reliable medical information which is key in determining the type, quality, and continuity of care a woman receives during pregnancy [3]. Comprehensive notes can also act as prompts to the healthcare provider highlighting what needs to be done (standards, referral criteria, and pregnancy high-risk features). Therefore, health system planners and other maternal health stakeholders must consider the development, updating and implementation of antenatal care records that are comprehensive (complete, appropriate, private, easily accessible, informative, and empowering) to address the key gaps that exist in the system.

**Fig. 1:**
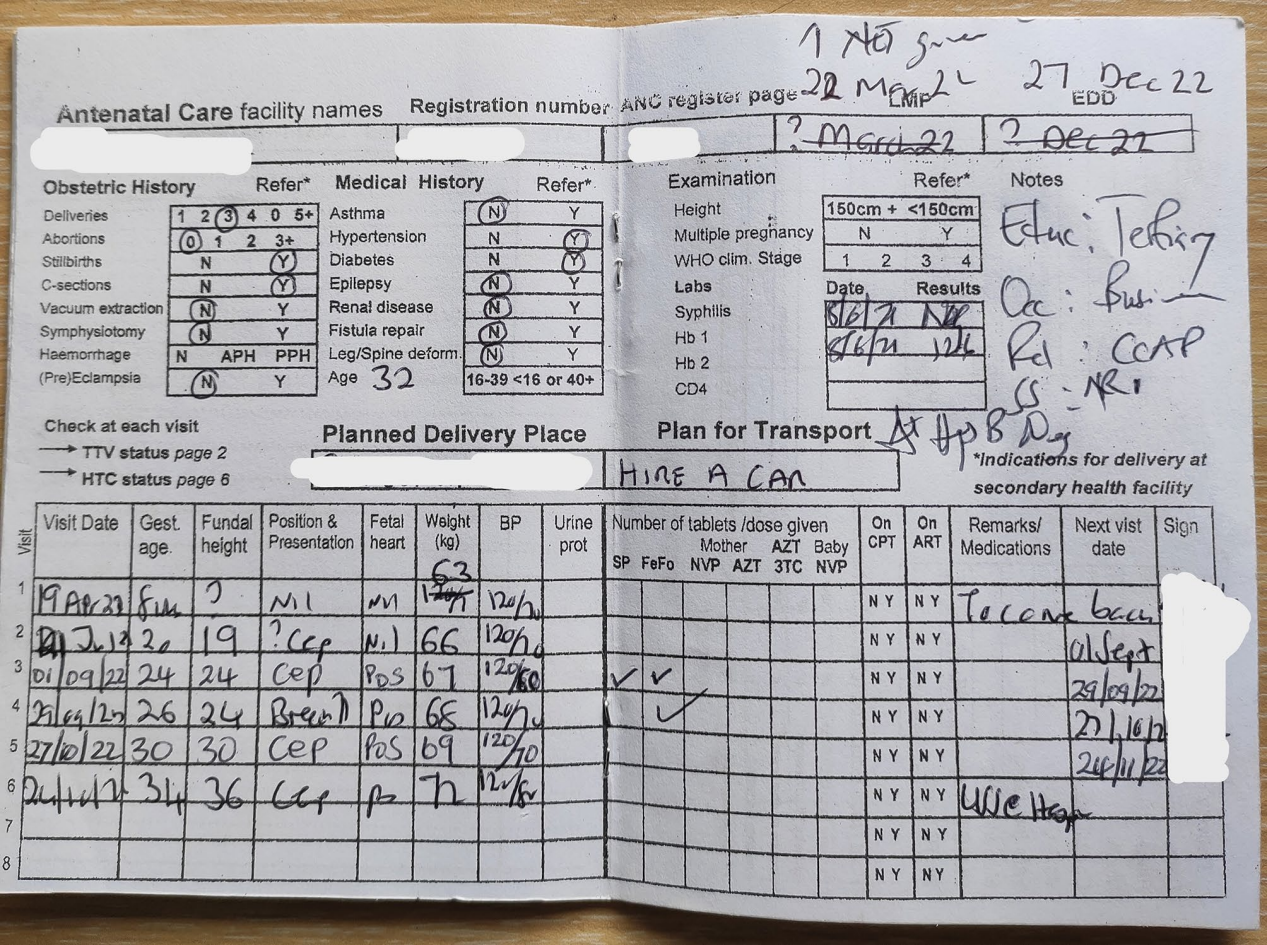
Centre page of the current woman’s health passport book. The centre page contains crucial ANC information for the woman. The figure clearly shows the documentation challenges that the current tool presents in the delivery of quality ANC

In Malawi, the woman’s health passport book is a paper-based tool that is used to document care pregnant women receive during their antenatal visits. It is designed to be portable and easy to carry, with pages dedicated to different aspects of maternal and child health, including family planning, past medical and obstetrical history, HIV testing and counselling (HTC), immunization, health education messages on birth preparedness, anemia, Tuberculosis, nutrition, and antenatal care visits. However, despite its intended purpose, the woman’s health passport book has several shortcomings. For example, it lacks comprehensive education content for pregnant women, clear instructions for healthcare providers on standard care and condition management, and does not fully reflect women’s rights or ensure the confidentiality of their health information. Additionally, the family planning section of the book does not reflect some of the newly available and highly utilized services e.g., subcutaneous depo-medroxyprogesterone acetate (DMPA-SC), while the HTC page lacks clear instructions on how healthcare providers should advise women and their partners. A key concern with the woman’s health passport book is the lack of space to document key aspects of antenatal care as recommended by the WHO 2016 guidelines on positive antenatal care, such as ultrasound scans, counselling, mental health checks, and tests to be done at each visit (Fig. 1). There are poor highlighters for referrals, comprehensive medication and prevention notes, nor space for a symphysis-fundal height graph. Furthermore, the tool is often reprinted by vendors without authorization, resulting in uncontrollable changes that undermine the comprehensiveness and clarity of the tool.

With Malawi’s commitment to improving the quality of antenatal care (ANC) (adopting the WHO 2016 ANC guidelines), the need to enhance tools and strategies in the delivery of ANC is necessary. The key WHO ANC recommendations adopted have to be reflected in current practice and current tools. This paper describes the development of an enhanced and contextualized woman hand-held case notes tool in a low-resource setting (Malawi). This would not only ensure compliance in practice but also aid in utilisation of tools and content that is feasible in our settings, thus significantly improving the delivery of quality care in Malawi.

## Methods

### Design

We applied a co-creative participatory approach, through three workshops, with key stakeholders to compare the current tool (ANC) contents to the WHO 2016 ANC guidelines, decide on key elements to be changed/added to the tool to improve adherence and change in practice, and redesign the tool to reflect the changes. The key stakeholders included obstetrics and gynaecology specialists, obstetrics and gynaecology registrars, clinical officers, nurse-midwives, district nursing and midwifery officers, safe motherhood coordinators, regulatory body/association representatives, and patient representatives. We also had one meeting with the National Safe Motherhood TWG where we presented the updated tool for their endorsement. Participatory approaches allow for inclusivity and ‘democracy’ in intervention development, thus being more appropriate for this work [5]. Co-creation accelerates the adoption and use of tools in practice because the critical stakeholders are involved and coideation and co-design of the intervention or tool [6].

### Setting

We conducted participatory workshops in two districts: Mangochi, eastern part of Malawi, and Dowa, central region of Malawi. The **first** workshop was conducted in Mangochi on 2 July 2022 because it was convenient for our team of researchers to meet the group of obstetrics and gynaecology specialists and registrars, who were having their 7th annual scientific national meeting in the district on that particular date. The **second** workshop was conducted on 21 July 2022 in Dowa district. The location was selected because it offered a central spot where invited stakeholders from all regions of Malawi would easily travel to and converge. For the same reason, the **third** workshop was also conducted in Dowa on 17 August 2022.

### Participants and sample size

**Fig. 2:**
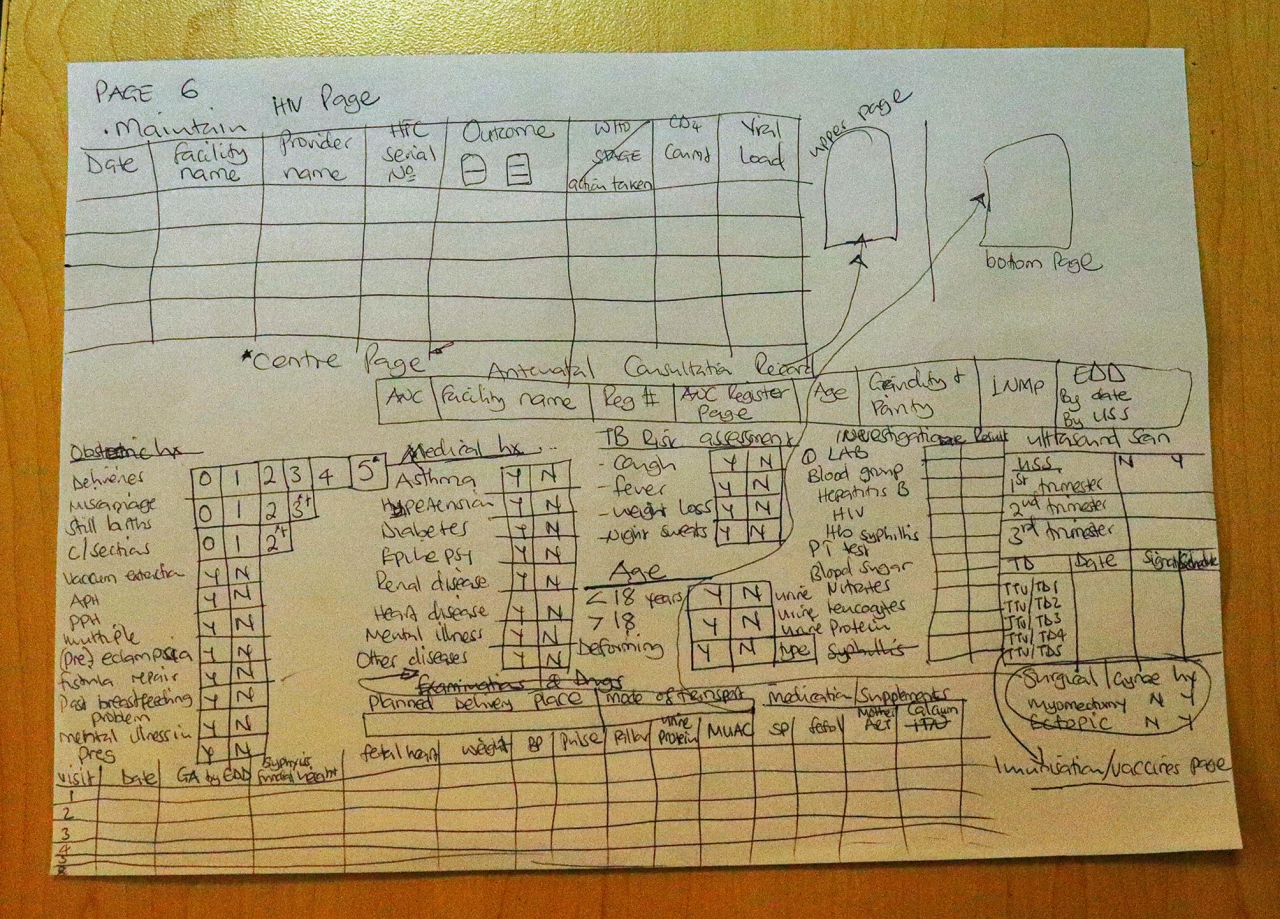
Sketch of some content and design of the new woman?s health passport book. The rough sketch outlines the ‘picture’ of how the participants want the passport book to look like. This was developed by participants during the third workshop in Dowa

In the first workshop, we conveniently included all participants who attended the workshop, while in the second workshop, participants were purposively selected based on their role in antenatal care services. Upon providing information to the prospective participants on the research aspects, they agreed to participate in the discussions and provide their input in developing the women’s hand-held case note tool. Obstetrics and gynaecology specialists and registrars were included as these are the key providers of specialist and high-quality care to pregnant women in the Malawian healthcare system. In the second workshop, the participants were mainly nurse-midwives and clinical officers (‘clinicians’), who are the primary providers of care in Malawi’s model of care at all levels; from primary health centres to tertiary facilities. The participants were drawn from facilities spread throughout Malawi to ensure geographical spread. We also had a patient representative during this workshop. The patient representative was a woman, teacher by profession. The value of involving a patient representative as a key end-user in tool development and health research has been well documented [7]. We included the Medical Council of Malawi as a regulatory body representative because they regulate the practice of health care workers by providing the scope of practice, influencing policy changes, and overall incorporating of new strategies in maternal health in Malawi. Finally, we included a graphic designer and experts from the government’s Central Monitoring and Evaluation Division (CMED) to better inform the tool re-designing during the third workshop (Fig. 2). The inclusion of CMED would also enhance the sustainability of the tool within government structures, as this is the central authority in designing healthcare delivery-related tools and other Health Management Information Systems (HMIS) in Malawi.

### Data collection

#### Focus group discussions

Prior to the workshops, prospective facilitators were trained in data collection methodology and familiarised with data collection tools and guide, during a preparatory session.

In the first co-creative workshop, participants were divided into groups of about five people, where they discussed the gaps in the current ANC tool, the barriers, facilitators, strategies, and what to include in the new ANC tool. Focus group discussions were conducted as defined by Wilkinson, 2004 [8]. Gaps were identified through a side-by-side comparison of Malawi’s Antenatal Care Matrix (Additional file 1, adapted from 2016 WHO ANC guidelines) and the current ANC tool, embedded in the woman’s health passport book. The matrix acted as a benchmark of the 2016 WHO ANC guidelines which essentially are supposed to appear in the ANC card and current practice. A trained facilitator facilitated the discussion using a discussion guide (Additional file 2). The facilitator also took short notes on the deliberations of the discussion. The facilitator did not influence the discussion in any way as the groups maintained autonomy by selecting their group leader, and taking their notes on flipcharts while engaging in the guided discussion.

In the second workshop (where we had a mixed bag of stakeholders), participants were divided into groups according to their roles. This helped in addressing any group dynamic issues that might arise when people of different cadres are put in a group to discuss differing lines of arguments. Every other procedure was the same as described in the first workshop. The third workshop involved redesigning of the tool by sketching on paper and interacting with the designer on key summarized aspects that have to be incorporated into the new ANC card.

Focus group discussions were most appropriate for data collection, because the type and range of data generated through the social interaction of the group are often deeper and richer than those obtained from one-to-one interviews, and it also examines in detail what the group members think and feel about the topic [9]. The discussions were recorded and then transcribed.

### Affinity diagrams

Upon completion of the within-group discussions, a representative of each group presented their key points under each topic to the whole audience. The topics included gaps in the current ANC tool (in comparison with the 2016 WHO ANC recommendations presented on the matrix), barriers to implementing a new improved tool, factors for optimal implementation of the improved tool, strategies for effective implementation, and key specifics to include or remove in the newly developed ANC tool. All the information collected was consolidated from all the groups into affinity diagrams (see additional files 3 and 4).

### Modified nominal group technique

The whole group (audience) of participants was then asked to prioritise five priority points under each topic (gaps, barriers, facilitators, strategies, and changes to the tools) from the consolidated data presented in the affinity diagrams. A facilitator led this session where he/she read out all the points under a topic and asked participants to vote the prioritized ones. Although the plan was to have five priorities under each subtopic, more than five were selected in all cases. The nominal group technique allows meeting participants to determine which issues require further, more in-depth inquiry and to draw attention to issues that may have been previously unidentified [10]. It therefore provides a great basis for prioritizing areas that need further attention without the interference of unbalanced involvement [10]. We adopted a modified nominal group technique because the participants had ample time to discuss the issues in a smaller group and were well aware of the contents. Again, the modified approach enabled us to quickly prioritise within the limited time constraints we had.

### Data analysis

Data were analysed through thematic analysis, and reduction and summaries in affinity diagrams. We followed an iterative approach as it fitted to ensure that the two ways of data analysis complement each other to enhance the meaning-making of the data. For the thematic analysis, we applied a six-step analysis approach as defined by Braun and Clarke, 2022 [11]. The steps include (i) Familiarising oneself with the dataset, (ii) Coding, (iii) Generating initial themes, (iv) Developing and reviewing the themes, (v) Refining, defining, and naming the themes, and (vi) Writing up. Three key members of the research team (LM, CK, ALNM) were more familiar with the data and created the initial codes upon reading few transcripts. The draft code list was further improved upon presentation to other team members. We ensured consistency of coding by the team members by putting in place checks. We then generated and developed themes, which underwent through refining by the whole team. The final list of themes was included in the write-up of the manuscript.

## Results

We had a total of 25 participants for the **first** participatory workshop in Mangochi, a total of 25 participants for the **second** workshop in Dowa, and a total of 8 participants in the **third** (tool re-design) workshop in Dowa. Tables 1 and 2, and 3 show a summary of the workshop participants for the 3 workshops respectively.

**Table 1:** Summary of participants for the first participatory workshop in Mangochi

Cadre	n	Facility/Organization
Obstetrics and Gynaecology specialists	1	Kamuzu Central Hospital
	3	Queen Elizabeth Central Hospital
	1	Private Clinic
	1	University of North Carolina project
Obstetrics and Gynaecology registrars	8	Kamuzu Central Hospital
	4	Queen Elizabeth Central Hospital
	1	Kamuzu University of Health Sciences
	1	Baylor /Area 25
	2	Mangochi District Health Office
Clinical Officers	2	Baylor/ Area 25
BSc Student	1	Kamuzu University of Health Sciences

**Table 2:** Summary of participants for the second participatory workshop in Dowa-Mponela

Cadre	N	Facility/Organization
Obstetrics and Gynaecology registrar	1	Lilongwe District Health Office
District Nursing Midwifery Officers	1	Lilongwe District Health Office
	1	Blantyre District Health Office
	1	Karonga District Health Office
Medical Officers	1	Lilongwe District Health Office
	1	Blantyre District Health Office
	1	Karonga District Health Office
Clinical Officers	1	Blantyre District Health Office
	1	Karonga District Health Office
Midwives	4	Karonga District Health Office
	3	Lilongwe District Health Office
	5	Blantyre District Health Office
Patient representative	1	Blantyre District Health Office
Associations	1	Medical Council of Malawi
Ministry of Health	1	Reproductive Health Directorate
	1	CMED

**Table 3:** Summary of participants for the third (tool re-design) participatory workshop in dowa-mponela

Cadre	n	Facility/Organization
Obstetrics and Gynaecology specialist	1	Queen Elizabeth Central Hospital
Resident Obstetrician and Gynaecologist	1	Lilongwe District Health Office
Regulatory bodies	1	Medical Council of Malawi
District Nurse-Midwife Officer	1	Karonga District Hospital
Nurse-Midwife Technician	1	Chilumba Rural Hospital
Ministry of Health representative	1	CMED
Senior Nurse-Midwife Officer	1	Area 25 Health Centre
Clinical Officer	1	Karonga District hospital

Five themes were identified in the analysis. These include (i) critical components missed in the current tool, (ii) reimagining the current ANC tool, (iii) opportunity for ultrasound scanning conduct and documentation, (iv) anticipated barriers related to the implementation of the newly developed ANC tool, and (v) cultivating successful implementation.

### Critical components missed in the current tool

Participants identified and reported crucial components that are absent or inadequately addressed in the current ANC tool in reference to the provision of quality ANC and effective implementation. These include the absence of sections and space for comprehensive personal, medical, surgical, and general history taking, a disorganised section for lab investigations, missing critical assessments such as TB risk assessment, missing health education content for mothers and pregnant women, lack of content on intimate partner violence and gender-based violence, missing counselling, and mental health checks, among others.

Okay then we can say that the gap now is that the commonly used health passport does not have most of the information on history taking… (Participant, Nurse -midwife, Dowa workshop).

Moving on to client education and counselling. This one is not there; we do not record it in the commonly used (health passport book) and a recommendation would be a designation of a special page for it… (Participant, Dowa workshop).

Participants stated that the current commonly used ANC card (health passport) presents key information-related challenges which hinder the provision of comprehensive quality care. Notably, the lack of space for comprehensive medical and surgical history was reflected as ‘dangerous’ and likely to affect labour procedures if the healthcare provider is not well aware of related past history on such in the health passport book.

I do not know what is in the (ANC) matrix, but in terms of the surgical history of a pregnant woman, I feel like there are special surgical cases for pregnant women that you have to consider when you are handling the pregnant woman for example if she had any surgical (procedures), was it just general? What am suggesting is perhaps to come up with key surgical conditions that can affect pregnancy in general enlist them and they… (Participant, Dowa Workshop).

Like the gynaecologist or indeed midwives can sit down (they know it is not every disease), they know key specific procedures that can affect pregnancy starting from antenatal up to labour delivery right…if I had surgery here (on any other place other than any connected to pregnancy-related surgery), it may not matter for me being a pregnant woman, but if I have got one previous scar like laparotomy or something that will affect bearing down in labour, those are the issues of concern… or maybe heart surgery, because in labour the heart has to function you need to bear down, so they can have that list and people will be able to know unlike the way it is (missing)… (Participant, Gynaecologist, Dowa Workshop).

For many participants, the gaps in the current available ANC tool were not only linked to the design of the tool and its content but also related to weak implementation policy. They reported the availability of multiple tools on the market that deviate from the standard one. This is due to printing and reprinting by small business owners, who prefer printing small-paged books for profit and omit critical content.

I think because of printing business people/printers are omitting some of these pages of information since they just want to make profits… (Participant, Dowa workshop).

In our group, we thought about regulating and the regulating body being the ministry, the ministry can find other people who can provide us with those books because if we want a standard document it needs to be regulated… (Participant, Dowa workshop).

### Reimagining the current ANC tool

Participants agreed on several changes to be made to the current tool to reflect the comprehensive nature of care and services required to be offered to the women. There was a consensus that the new tool should improve based on the already existing one. Some of the changes agreed upon included a change in size from the current A6 to A5, adding more pages to have adequate space for an antenatal woman’s personal history, surgical history, and general history, maintenance of the yellow colour, and updating information on the cover page to reflect reminders to the woman to always bring the health passport book to the facility when pregnant. Participants reported on the need to include a respectful maternity care charter. Further, participants agreed that each passport book should have space to accommodate information on about 3 pregnancies, a change from the current book which only has space for single pregnancy (Table 4).

**Table 4:** Summary of some of the suggested changes in the old woman’s health passport book and rationale for making the change

Some of the Suggested Changes	Rationale
Size from A6 to a standard A5	To accommodate more pages and adequate space for antenatal women’s personal history, surgical history, and general history. *“We suggested A5 because we were considering how much information we are going to put on the middle page, the number of columns because we were thinking that being a summary it has to have several columns and some readings.”* **Participant, Dowa workshop** *“… from experience, we are very poor in going back and checking what others have written so in our group we discussed maybe it has to be an A5 so that the most important information should be on the centre page because that’s the page which most health workers review, but flipping into other pages most of the health workers they don’t, so we might miss some of the important information. And if we had more pages and bigger size, maybe us healthcare workers will now be interested in checking on the other pages…’’* **(Obs and Gynae specialist, Mangochi Workshop)**
Information on the cover page and educational content for pregnant women	To reflect reminders to the woman to always bring a health passport whenever she is visiting a facility and flag out key danger signs in pregnancy that should prompt a woman to seek medical attention
Respectful maternity care charter	To remind clients of their rights and to prompt health care providers to provide care by the rights of women and their babies
To include 3 ANC center pages	To include at least information for 3 pregnancies. *“Maybe they should increase the number of center pages so that there is space to include the history of at least 3 pregnancies just like in South Africa, they have space for all the previous pregnancies a woman has had, so it is easy to follow.”* **Participant, Mangochi workshop**
Add more pages	To have adequate space for comprehensive antenatal woman’s personal history, surgical history, and general history “…*should increase the number of pages to incorporate the information that has been updated to accommodate the recommendations from the gaps”* **Participant, Dowa workshop**

Participants reported the need to include comprehensive assessments, updated drug administration (e.g., the inclusion of calcium supplementation to the medicine/preventive measures section), fetal measurements, tests such as syphilis test, and health talks and education content throughout pregnancy, in reflection to the WHO’s 2016 ANC guidelines. Despite the resource and implementation challenges our healthcare system faces, there was still a consensus that the updated tool should be ambitious and show components that if resources are available, comprehensive quality care provision will be initiated.

Before we proceed, somewhere you were reading about calcium and some people said we should not include calcium as one of the supplements because it is not given to every woman who is pregnant, I think it should be included if we have the resource, if we have the drug we need to supplement our women considering our large percentage who are becoming pregnant are above 30, above 35 who are at risk of pre-eclampsia, so I think giving calcium pre or during pregnancy we can reduce cases of pre-eclampsia in our women, if we have the capacity we can do that… (Participant, Dowa workshop).

The current woman’s health passport book reflected old recommendations about HIV testing and counselling and family planning, thus the participants agreed that the new tool being developed should reflect the ‘Test and treat’ HTC approach as well as updated family planning content as per updated guidelines. The participants proposed the inclusion of key reminders on the schedule of critical immunization e.g., tetanus toxoid vaccine (TTV) schedule to have dates of the next dose as reminders. They also reported the need to include a section of ‘other vaccines’ to reflect newly developed vaccines such as COVID-19 vaccine status.

#### Opportunity for ultrasound scanning conduct and documentation

There was a perception from the participants that prevention and treatment of preterm births would require accurate assessment of gestational age during pregnancy through ultrasound scanning; a component currently missing in the present healthcare passport book (ANC tool). The WHO endorses one ultrasound scan before 24 weeks gestation for all pregnant women [1]. Participants reported that the lack of ultrasound scanning results significantly increased complications of preterm labour in Malawi.

We also have the issue on ultrasound…remember in the conversation there was a mention that it needs to be added somewhere to determine gestational age whereby someone doesn’t know her normal menstrual…so that tool is missing right? It would significantly help in reducing cases of preterm births, and unplanned labour from happening. We need it, it shows the WHO recommendations which we have adopted… (Participant, Mangochi Workshop).

Many participants were of the view that the component of ultrasound scanning should be prioritised in the new ANC tool and should have its separate page. Space should be adequate for 3 scans throughout pregnancy, for all needed measurements, following WHO recommendations. The estimated date of delivery (EDD) should be categorised as through scanning or the Last Normal Menstrual Period date. Thus, there was a recommendation to further increase the size of the booklet size.

So here space for EDD should be grouped by date and scan…we should take into consideration both. We can, for example, have 3 scans per pregnancy. In scanning we are looking for many things, that is gestation, we also look at how many kids are, many measurements… so on the findings, we need to incorporate all that in the space in the book… (Participant, Mangochi Workshop).

Ultrasound scan by recommendations is supposed to be done three times so there was a question on recommendations that can’t we put a space for frequency somewhere… It’s first trimester, second trimester, and third trimester scan…so to simplify space I was thinking can’t we have an antenatal space for all 3…? (Participant, Dowa Workshop)

Few participants felt that the addition of content on ultrasound scanning (an entirely new component not available in the old health passport book) in the newly developed women’s health passport book would present a challenge in the implementation of the tool due to regulatory and ethical considerations that need to be considered for the operationalisation of the service. However, many participants were of the view that leveraging on the existing stakeholders and partnerships working in ultrasound scanning space would present a solution to expedite and operationalise the development of guidelines, pieces of training and capacity building, task shifting, and sustainability, thus positively impacting implementation.

Partners are already there. But on ethical and legal environmental factors…where those that have been trained need to be recognized by regulatory bodies. Trained personnel should be involved in guideline formulation and capacity building… like training others. Maybe this will ensure that this is not just a project but a national thing… (Participant, Dowa Workshop).

### Anticipated barriers related to the implementation of the newly developed ANC tool

#### Limited resources

Many participants reported a lack of/inadequacy of essential resources such as drugs and medications, test kits, and ultrasound machines, as a key implementation barrier to effectively offer services stipulated in the newly developed ANC tool and policy guidelines.

We have discovered that there are some tests that are not done at a certain level because of the unavailability, I can say maybe the policies or the way our guidelines are…. If we take things like sugar, they don’t have equipment’s like glucometer, glucose kits, so availability of resources is also a barrier in the implementation of some of the tools… (Participant Dowa Workshop).

The other barrier is the continuous stockout e.g., Calcium (which we are supposed to provide to clients), is not on central medical stores purchase list. Budget to this is very little… (Participant Mangochi Workshop).

Participants also highlighted how the unavailability of sustained healthcare worker training mechanisms and capacity building after updated guidelines affect the continuation of quality care provision in Malawi’s healthcare system. There was a strong feeling that this could affect the optimal implementation of the newly developed tool.

Ok so on resource barriers, the first one that we wrote, that is talking about health facilities not being able to provide health passports, implementation barrier could be the training or orientation barrier that we were talking about, and resource barrier could be where the government does not make the health passports available for people to purchase from them, the authentic health passports (Participant, Dowa Workshop).

Some participants reported that there is a need to rethink the attitude of some healthcare providers towards documentation; they present negligence in their conduct of work through poor documentation thus affecting continuity of quality care throughout a woman’s pregnancy journey. Other participants felt that policies and guidelines in Malawi are weak, thus regulatory capabilities such as centralised printing of standard health passport books, are mostly met with challenges. Financial allocations towards health are also limited, hence stockout of books, essential medications/preventive measures are very common.

On barriers, on gender-based violence, WHO came up with a tool on Intimate partner violence, but a lot of healthcare workers have not been trained to identify at risk groups. And people (Healthcare workers) do not bother to ask. This even happens in the private facilities… (Participant, Mangochi Workshop).

### Cultivating successful implementation

Participants reported several key recommendations to be considered for optimal implementation and utilisation of the new tool in the quest to attain quality antenatal care in Malawi. Participants felt strongly about the need for the government to put in place strict measures to ensure that the printing of the health passport book is regulated. They strongly recommended that this should either be centrally regulated or decentralised to the district council level. With regulated printers in place, issues of the tool deviating from the currently developed standard would be addressed.

We can put it (printing) at level, it should come at council…we can do it as a district, us health personnel at council we can print these because if we push this to the ministry, they always say it will not pass…so maybe the director at the council can print these. Printing rights should be at council level to maintain a standard document… (Participant, Dowa Workshop).

Participants also recommended the need for political will and proper management in relation to financing and pushing the agenda of the newly developed tool. They strongly felt that the amount of money allocated for health activities remains very low and that management hiccups across levels of government and the healthcare system make achievement of the health needs of the country a daunting task.

15% (as a total form of government expenditure) is small, we also don’t manage… (Participant, Dowa Workshop).

.resource barrier could be where the government or management does not make the health passports available for people to purchase from them the authentic health passports. This could also happen with the new passport books if the financing is not increased… (Participant, Dowa Workshop).

In addition to the changes suggested to be made in the new ANC tool, participants suggested that there is a need to add components of client counselling across the pregnancy journey, comprehensive health talks coupled with education content for the mothers on eating healthy, maintaining positive mental well-being, and identifying key danger signs that would warrant them to rush to their nearest healthcare facility. However, they recommended the need to have capacity-building activities to fully implement the changes and tally with the changes and updates in the guidelines.

**Fig. 3:**
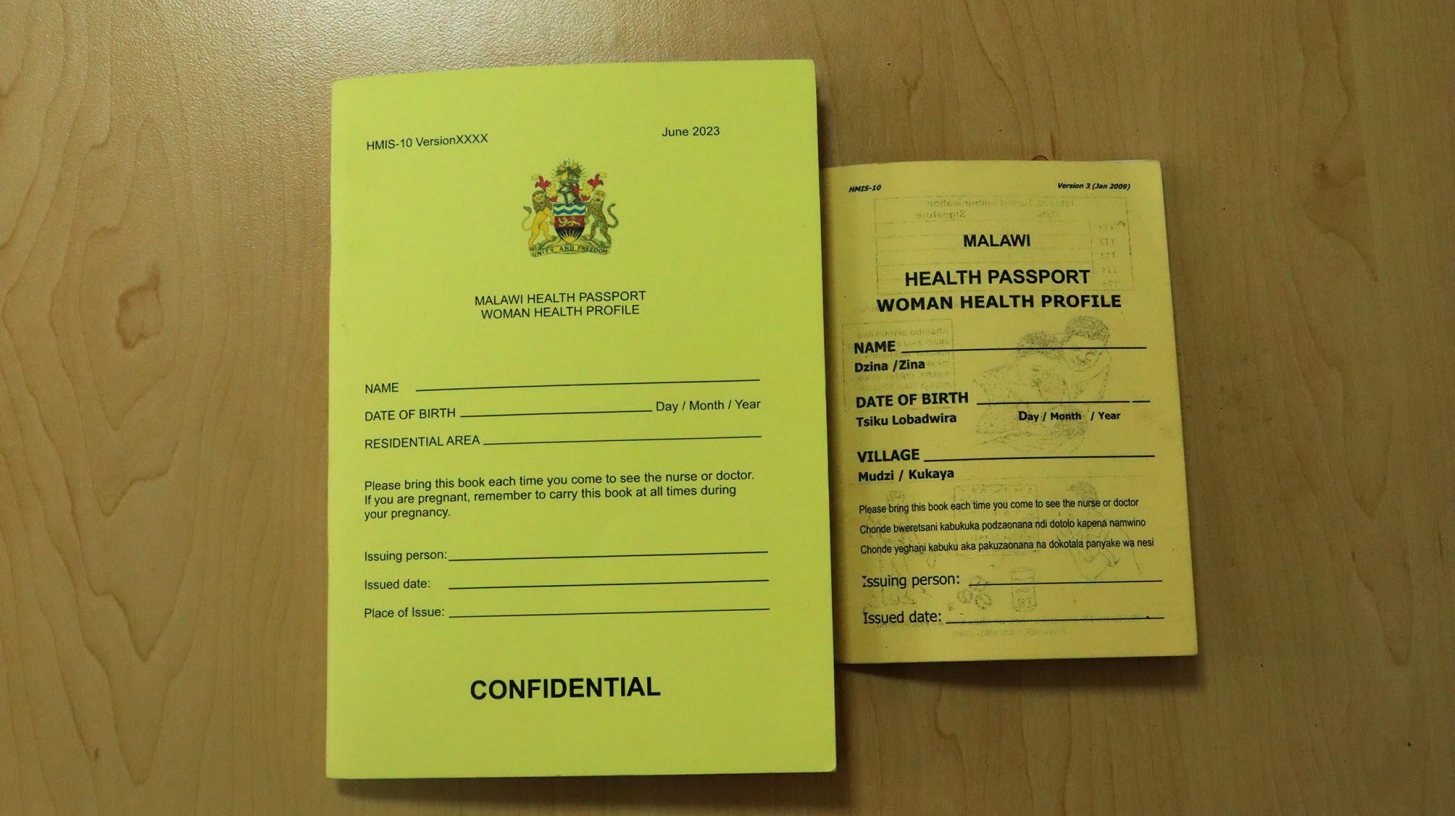
The newly developed handbook (left-hand-side of the reader) versus the old handbook (right-hand-side of the reader)

The newly developed handbook is bigger and has comprehensive information relating to ANC services, key highlighters prompting the provider on what to do, including referral criteria and high-risk features and assessments (see additional file 5 and Fig. 3). The old passport book is small and presents challenges to healthcare providers in documenting relevant ANC information among other services (see Figs. 1 and 3). Table 5 summarises some of the comparative aspects of the old woman’s health passport book and the newly developed woman’s health passport book.

**Table 5:** Comparison of some aspects of the old woman’s health passport book Vs Newly developed woman’s health passport book

Aspect of comparison	Old woman’s health passport book	New woman’s health passport
Size and Page	A6 size, very few pages	A5 size, increased number of pages
Color	Yellow	Yellow (Maintained the color to avoid confusion because people are already used that this color is for women of childbearing)
General history page	Limited content on personal history, family history, past medical and surgical history	Personal details, family history, past medical, surgical, and vaccination history with some additional information on each section
First page	Limited Health Education information	Respectful maternity care charter
Second page	General History	Educational content for pregnant women
Vaccination/ immunization information	Tetanus Toxoid vaccine schedule only	Tetanus toxoid/tetanus-diphtheria vaccine schedule with key reminders on dates for the next dosage, and space for any other vaccines information (e, g COVID-19 vaccines)
HIV testing and counselling page	Date, facility name, HTC serial number, Outcome, and provider’s signature	Date, facility name, HTC serial number, Outcome, provider’s signature, and additional information such as action taken/recommendations, viral load, and CD 4 count to make sure all information about HIV is on one page
ANC centre pages	2 pages only, with inadequate information on USS, Infection risk assessment, and history of the woman	3 comprehensive pages to have information for at least 3 pregnancies in one passport book with comprehensive antenatal care information such as TB risk assessment, EDD by USS, counselling, surgical/gynecological history, and laboratory investigations. A comprehensive outline of preventive measures and health talks across the 3 trimesters and 8 visits respectively. Comprehensive demographic history, obs/gynae history, and history of mental health conditions during past pregnancy. All information added together with key highlighters/prompts/reminders to the healthcare provider for referral
Other	No space for Ultrasound scanning information	Ultrasound scanning details page for documentation of at least 3 scans per pregnancy, with a wide range of measurements outlined.
		Added international symphysis fundal height standards page (graph)
		Checklist after immediate childbirth

The newly developed woman’s hand-held passport book was endorsed for piloting and facility rollout by the National Safe Motherhood Technical Working Group in Lilongwe, Malawi (see additional file 5 for the whole digital health passport book).

Additional files 3 and 4 show the axillary analyses summaries and reductions of the two key participatory workshops (Mangochi on 2 July 2022 and Dowa on 22 July 2022 respectively).

## Discussion

Utilising feedback from key maternal health stakeholders, we developed comprehensive woman-hand-held case notes in the quest to improve the quality of antenatal care in Malawi. There was a general understanding among participants that the current woman’s health passport and antenatal care sections reflected numerous gaps that would act as bottlenecks to the efforts in the provision of quality care to pregnant women and women in general in Malawi as per the WHO 2016 ANC guidelines, which we adapted as a country. Participants deliberated on the following; adding education content for the women, inclusion of updated preventive measures and medications, changing size of the passport book from A6 to a standard A5, and exploring opportunities to normalise ultrasound scanning for dating pregnancy. There was a general understanding on anticipated challenges to implementing the changes, which included resource mobilisation, political will and healthcare provider-related challenges.

Of key highlight, participants noted the need to include education content for mothers, so that mothers can become knowledgeable on the critical times they should seek care during their pregnancy journey (e.g., when bleeding, when they have headache, or when they experience or at risk of different forms of gender-based violence). Efforts to enhance antenatal education for pregnant mothers in Malawi have been reported before [12]. However, there is a dearth of literature on the inclusion of separate education content and reminders in women’s case notes, which our current work addresses. Elsewhere, studies have reported a positive impact of structured antenatal care education on alleviating anxiety, and maternal depression and enhancing maternal self-efficacy [13–15]. A comprehensive global literature review on approaches to delivering information to mothers reported that the inclusion of information on leaflets was effective and significantly improved mother’s self-efficacy and positive pregnancy experience [16]. It was reassuring to have participants reach a consensus to include content on what will happen during each ANC visit, and health talks to ensure adequate information to the pregnant women. There was a general agreement that respectful maternity care should be included hence the need to lay out the respectful maternity care charter [17] in the new book.

Participants were of the view that the current stipulations related to medications and preventive measures presented a gap; they did not include key updated supplements (e.g., calcium) and regimens of key treatments such as antiretroviral therapy (ART). In calcium deficiency populations, the WHO has recommended supplementation of calcium in pregnancy to reduce preeclampsia severity, overall maternal morbidity, and neonatal mortality [18]. Similarly, the health governing body has stipulated an update of antiretroviral therapy [19], which our women’s health passports do not reflect such. Hofmeyr et al., 2018 [20], in a comprehensive systematic review, reported a reduced incidence of pre-eclampsia and hypertension, and related morbidity and mortality, in women who received a relatively low dose of calcium supplementation in a generally calcium-deficient population. Lacking such supplementation would result in severe morbidity, mortality, and premature births. In view of this, there was a general understanding of the need to make changes and add key prompters to the provider on what to do, content to reflect recent standards, referral criteria, and even high-risk features. Stakeholders working in the scope of maternal HIV advised on the key updates related to HTC and ART which now reflect in the new health passport book.

It was reassuring to see many stakeholders highlight the need to normalise ultrasound scanning and the inclusion of documentation sections in the health passport book. Although nearly universal in developed countries, obstetric ultrasound scanning remains scanty and not integrated into routine services in most developing nations [21]. There has been, however, notable progress in the developing world as nations are now steadily integrating obstetric ultrasound scanning into their routine healthcare practice. In Vietnam, for example, one ultrasound scan during pregnancy is universal [22]. In India, over 60% of pregnant women received at least one ultrasound scan in 2016. To achieve this, there have been significant investments in resources and building capacity of healthcare workers to normalise obstetric ultrasound scanning. Elsewhere, healthcare workers are motivated and driven by the belief that they can’t do without an obstetric ultrasound scan during pregnancy management [23]. Similarly, in our work, participants perceived ultrasound scanning as a key intervention that every woman should undertake to correctly date her pregnancy and equally prevent the risk of preterm delivery and other pregnancy-related complications. These deliberations are in accordance with the WHO 2016 ANC guidelines [1]. The new health passport book therefore has incorporated dating of pregnancy through ultrasound scans and the last normal menstrual period (LNMP).

Participants felt that the current health passport book was small. Therefore, an increase in size was a necessity to directly have adequate space for comprehensive history taking of the woman, post-delivery reminders and checklist, the inclusion of critical risk assessments (e.g., TB), robust lab investigations and documentation, counselling and mental health checks, International Symphysis-Fundal Height Standards, health talks, and updated HIV and family planning pages. However, the participants also cautioned that the passport book should not be too big, thus a change from A6 to A5 was agreed. In South Africa, a much bigger newly developed maternal case record was perceived as optimal for documenting information, but too big to handle and care for [24]. Sibiya et al., (2015), attributed the challenges of creating an ‘optimal-sized’ tool to the lack of involvement of key stakeholders (such as midwives and other healthcare providers) in the development and design process of the tool [24]. In our study, we ensured that relevant stakeholders were fully involved in the development of the tool. We also considered safe storage aspects of the tool, as an A5-sized passport book can easily fit in a plastic bag (such as a 1 kg empty plastic sugar packet), preventing it from getting wet during the rainy season. It is important to have a more comprehensive, optimally sized women’s health passport book. A comprehensive systematic review reported that women were satisfied with the quality of care they received when they carried their own adequate and comprehensive case notes/health passport books [2]. In Japan, having a comprehensive health passport book was associated with quality of care indicators such as assisted delivery by trained personnel, adherence to immunization schedule, and receiving of ‘satisfactory’ maternal care, among other advantages [25].

Participants understood that there could be challenges with the implementation of the newly developed tool such as resource mobilization, healthcare provider-related challenges with documentation and provision of more services, and political will. Malawi’s budget is heavily donor-dependent and the World Bank reported that Malawi’s healthcare expenditure is at just 2.4% of the Gross Domestic Product [26]. Malawi’s healthcare system fails to adequately provide free primary health care due to a relative inadequacy of financing mechanisms in relation to need. Over a quarter (27%) of financing comes from private sector, and from this, over 53.4% is from out of pocket [27]. These factors present challenges that would impede optimal implementation and roll out of the newly developed tool. Participants, however, were positive that it is still a possibility to implement the tool within the context. They recommended strengthening already existing policies to ensure that printing and distribution of the standard health passport is done by the government, increased resource allocation by the government for investment in drugs, equipment, and healthcare infrastructure, strengthening public-private partnerships, and continued capacity building of healthcare providers to ensure that their skill-sets are up-to-date as updates are made in the tools to reflect a change in health service provision.

Of key strength in this study, we found our participatory approach (group discussions and modified nominal group technique) as effective and efficient way of soliciting information from multiple stakeholders and developing a final woman’s health passport book (ANC tool). The process ensured that all stakeholders were fully engaged in the discussions and reached a consensus on the changes made. Nelson et al., (2022) found that the nominal group technique was the most appropriate method for physicians to identify and priorities key elements of a burnout prevention tool and intervention in California, USA [28]. The participatory approach also offers flexibility for use within groups of different capacities as reported in a systematic review on the use of the nominal group technique in a population of individuals with differing cognitive abilities [29]. Generally, our participatory approach exhibited an active involvement of stakeholders, which is highly recommended within the scope of tool/intervention development [5].

Further, to our knowledge, this work is the first in Malawi that has attempted to develop comprehensive woman hand-held case notes, utilising a participatory approach that engaged all key stakeholders in the field of Antenatal care and women’s health. The output (updated health passport book) has been endorsed by the National Safe Motherhood Technical Working Group (the central authority) for implementation and piloting in Malawi’s healthcare system. We are the first to explicitly highlight the processes and procedures followed in this work so that other entities can learn from it.

The major limitation of the work is that it engaged mostly healthcare providers and had limited representation from the patient representatives. This means the recommendations and accounts are hugely based on the care providers and not necessarily accounts from pregnant women. The only patient representative we had was a non-pregnant learned participant. It would have been better to have an illiterate pregnant woman, to understand her expectations and definitions of quality antenatal care. However, we are of the view that the majority of the changes in the book were technical changes, which would be best addressed with information and feedback from technical people in the space of antenatal care and women’s health. The next phase of the work, which will test the acceptability of the tool, will engage a wider range of end-users (patient representatives). The tool will be translated in multiple languages and made in formats that will allow this testing.

Our work has implications for current practice in the provision of comprehensive antenatal care in Malawi. We need resource investments to ensure that the resources stipulated in the tool are available at all times. Women need to be empowered and educated on their rights and demand better quality care. Healthcare workers’ capacity should continuously be enhanced so that their skills match with the updates in guidelines. The government should strengthen legal, ethical, and policy space to avoid deviations from the current tool; this also translates to the government increasing its resource allocation to the health sector in Malawi.

## Conclusion

Efforts to attain comprehensive antenatal care and achieve goals of universality in quality healthcare are possible only if tools in the healthcare system reflect the updates and changes stipulated in globally adopted or adapted guidelines. Our efforts reflect a pioneering attempt in Malawi to improve women’s hand-held case notes, which we know help in enhancing the quality of care and improve overall women’s satisfaction with their healthcare system. Further work in Malawi should be to pilot and then evaluate the effectiveness and acceptability of the newly developed hand-held passport book in the provision of quality care in our antenatal clinic.

### Electronic supplementary material

Below is the link to the electronic supplementary material.

Supplementary Material 1

Supplementary Material 2

## Data Availability

Data and related materials for this work are available upon reasonable requests to the corresponding author.

## Abbreviations

ANC: Antenatal care
ART: Antiretroviral therapy
BSc: Bachelor of Science
CMED: Central Monitoring and Evaluation Division
EDD: Estimated date of delivery
GDP: Gross Domestic Product
HIV: Human Immuno-deficiency Virus
HTC: HIV testing and counselling
LNMP: Last normal menstrual period
TB: Tuberculosis
TTV: Tetanus Toxoid Vaccine
TWG: Technical Working Group
UK: United Kingdom
USA: United States of America
USS: Ultrasound scan
WHO: World Health Organisation
