# Supplementary material for: Developing comprehensive woman hand-held case notes to improve quality of antenatal care in low-income settings: participatory approach with maternal health stakeholders in Malawi
Source: BMC Health Serv Res. 2024 May 15;24:628. doi: 10.1186/s12913-024-10922-3 (PMC11094996; doi:10.1186/s12913-024-10922-3)
Supplement: Supplementary file 1 — Supplementary Material 1 [file 12913_2024_10922_MOESM1_ESM.docx]

**Additional Files**

**Additional File 1. Malawi Antenatal Matrix: Adapted from WHO 2016 ANC Guidelines**

|  | | | | | | | | |
| --- | --- | --- | --- | --- | --- | --- | --- | --- |
| **Gestational** | **First** | **Second Trimester** | |  | **Third**  **trimester** | |  |  |
| **Number of Contact** | **1** | **2** | **3** | **4** | **5** | **6** | **7** | **8** |
| **Parameter*** | **up to 12 wks** | **20 wks** | **26 wks** | **30 wks** | **34 wks** | **36 wks** | **38wks** | **40 wks** |
| **Registration** | √ |  |  |  |  |  |  |  |
| **Comprehensive history-taking** |  |  |  |  |  |  |  |  |
| Personal history | √ |  |  |  |  |  |  |  |
| Family History | √ |  |  |  |  |  |  |  |
| Social History | √ |  |  |  |  |  |  |  |
| Past medical surgical/history | √ |  |  |  |  |  |  |  |
| Past obstetric history | √ |  |  |  |  |  |  |  |
| Past breastfeeding history | √ |  |  |  |  |  |  |  |
| History of current pregnancy | √ |  |  |  |  |  |  |  |
| Risk assessment for TB | √ |  |  |  |  |  |  |  |
| Mental Health History & assessment | √ | √ | √ | √ | √ | √ | √ | √ |
| History on GBV/ IPV | √ | √ | √ | √ | √ | √ | √ | √ |
| **History of complaints in current pregnancy** | √ | √ | √ | √ | √ | √ | √ | √ |
| **Observations and clinical investigations** |  |  |  |  |  |  |  |  |
| Blood pressure | √ | √ | √ | √ | √ | √ | √ | √ |
| Weight | √ | √ | √ | √ | √ | √ | √ | √ |
| Height | √ |  |  |  |  |  |  |  |
| Gait | √ |  |  |  |  |  |  |  |
| MUAC | √ |  |  |  |  |  |  |  |
|  |  |  |  |  |  |  |  |  |
| **Physical examination** |  |  |  |  |  |  |  |  |
| Head-to-toe including: | √ | √ | √ | √ | √ | √ | √ | √ |
| Pallor | √ | √ | √ | √ | √ | √ | √ | √ |
| Pedal oedema | √ | √ | √ | √ | √ | √ | √ | √ |
| Breast examination | √ |  |  | √ |  |  |  | √ |
| Obstetric examination |  |  |  |  |  |  |  |  |
| Fundal height | √ | √ | √ | √ | √ | √ | √ | √ |
| Foetal poles/lie |  |  | √ | √ | √ | √ | √ | √ |
| Foetal presentation |  |  | √ | √ | √ | √ | √ | √ |
| Engagement of presenting part |  |  |  |  | √ | √ | √ | √ |
| Foetal heart sounds |  | √ | √ | √ | √ | √ | √ | √ |
| Vulval inspection and soft tissue assessment (genital ulcers, vaginal discharge, bruises, genital mutilation) | √ |  |  |  |  |  |  |  |
| Bony pelvis assessment (cephalo-pelvic relationship) |  |  |  |  |  | √ |  |  |
| **Laboratory investigations and Ultrasound** |  |  |  |  |  |  |  |  |
| Blood |  |  |  |  |  |  |  |  |
| Haemoglobin | √ |  |  |  |  | √ |  |  |
| Grouping and rhesus factor | √ |  |  |  |  |  |  |  |
| HTC | √ |  |  |  | √ |  |  |  |
| syphilis testing | √ |  |  |  | √ |  |  |  |
| Hepatitis B testing | √ |  |  |  | √ |  |  |  |
| Ultrasound | √ | √ |  |  | √ |  |  |  |
| Urine |  |  |  |  |  |  |  |  |
| Protein | √ | √ | √ | √ | √ | √ | √ | √ |
| Sugar | √ | √ | √ | √ | √ | √ | √ | √ |
| Acetone | √ | √ | √ | √ | √ | √ | √ | √ |
| Nitrate | √ | √ | √ | √ | √ | √ | √ | √ |
| Leukocytes | √ | √ | √ | √ | √ | √ | √ | √ |
| TB testing (if applicable) |  |  |  |  |  |  |  |  |
| Pregnancy test | √ |  |  |  |  |  |  | |
| **Drug administration, supplementation, and immunization** |  |  |  |  |  |  |  |  |
| Iron | √ | √ | √ | √ | √ | √ | √ | √ |
| Folic acid | √ | √ | √ | √ | √ | √ | √ | √ |
| Tetanus toxoid | √ | √ |  |  |  |  |  |  |
| Albendazole |  | √ |  |  |  |  |  |  |
| IPTp-SP (or other antimalarial) |  | √ | √ | √ | √ |  | √ |  |
| Calcium daily supplementation (1.5-2.0 gr oral element) For high risk group for preeclampsia |  | √ | √ | √ | √ | √ | √ | √ |
| Food Supplementation | √ | √ | √ | √ | √ | √ | √ | √ |
| LLITN distribution | √ |  |  |  |  |  |  |  |
| **Client education and counselling** |  |  |  |  |  |  |  |  |
| - Process of pregnancy and minor disorders | √ | √ |  |  |  |  |  |  |
| - Diet and nutrition | √ | √ | √ | √ | √ | √ | √ | √ |
| - Rest and exercise in pregnancy | √ | √ | √ | √ | √ | √ | √ | √ |
| - Personal hygiene | √ | √ | √ | √ | √ | √ | √ | √ |
| - Danger signs in pregnancy | √ | √ | √ | √ | √ | √ | √ | √ |
| - Use of medicine in pregnancy | √ | √ | √ | √ | √ | √ | √ | √ |
| - Effects of STIs/HIV | √ | √ | √ | √ | √ | √ | √ | √ |
| - Exclusive breastfeeding | √ | √ | √ | √ | √ | √ | √ | √ |
| - Symptoms/signs of labour |  |  | √ | √ | √ | √ | √ | √ |
| - Plans for delivery (birth preparedness) | √ |  | √ | √ | √ | √ | √ | √ |
| - Information on postpartum period and care | √ | √ | √ | √ | √ | √ | √ | √ |
| - Family planning | √ |  |  | √ | √ | √ | √ | √ |
| - Harmful habits (e.g., smoking, drug and substance abuse, alcoholism) | √ | √ | √ | √ | √ | √ | √ | √ |
| - Schedule of return visits | √ | √ | √ | √ | √ | √ | √ | √ |
| - Counselling on correct application of FE tablets (and others) and of taking SP or another Antimalaria | √ | √ | √ | √ | √ | √ | √ | √ |

* wks = weeks

**Additional file 2. Group discussion guide**

| Component 1  **Gaps Identification** | Identify items that are missing on the ANC card but need to be included per WHO guidelines |
| --- | --- |
| Component 2  **Tool** | Suggest ANC Tool that need to be developed/revised to include your items identified above.  For every tool mentioned, suggest components  What specifications should the tool have?   - Size e.g., pocket size, A4, A5 etc - Colour - How big? e.g., number of pages, page flow |
| Component 3  **Barriers** | What are potential Barriers to development/revision and implementation of ANC tools?   - Probe on management barriers, implementation climate barriers, Resource barrier and implementation resources barrier |
| Component 4  **Facilitators** | What could be the facilitators to implementation of a new tools in practice?   - Probe on readily available resources, policies, etc that can advance the implementation. |
| Component 5  **Strategies** | What strategies should be put in place to facilitate and sustain the implementation of a new/revised tool in practice? |
| **NOMINAL GROUP TECHNIQUE** | |
| List of selected items  Priority Setting | Prioritise items to be included in the ANC tool_Consesus methodology. |

**Additional File 3. Dowa workshop Affinity diagram/reduction summary for whole group discussion.**

**Facilitators of implementation of the newly developed women’s health passport book**

| **Gaps Identified in the current tool (Women’s health passport book/ANC card)** | **Changes to be made to the women’s health passport book.** | **Barriers to implementation of the newly developed women’s health passport book** |  | **Strategies to optimally implement the new women’s health passport book.** |
| --- | --- | --- | --- | --- |
| -  Current ANC tool does not have demarcation of trimesters  -No personal history, social and family  -No past breastfeeding history  -Incomplete history of current pregnancies  -No TB risk assessment  -No mental health assessment  -No history of GBV/IPV  -No observations of MUAC  -Inadequate space for recording findings  -No grouping and rhesus factor, Hepatitis B  -No ultrasound  -Lab investigations i.e Sugar and Leucocytes, acetone, nitrate, and pregnancy test  -Space for documenting albendazole  -Space for documentation of drugs e.g albendazole, LLITNs  -No client education and counselling  -Number of caesarian sections on obstetrics history  -History of fistula repair  -No chlonological order  -Missing important information, e.g. heart problems  -No space for pallor, pedal edema, and breast examination  -Vulva inspection and soft tissue inspection  -Calcium and food supplement  -No space for physical examination assessment  -Consider Covid-19 vaccination  -Engagement of presenting part  -Include all vital signs  - | 1. Size should be A5 2. Add more pages 3. Counselling page 4. Space for Centre pages be at least three pages 5. Mental health assessment 6. GBV and IPV 7. Good quality paper like under 5 passport 8. Maintain yellow colour cover page 9. TTV schedule to be included clearly 10. Two Centre page (times 2) 11. Space for scanning results 12. Calcium and food supplements 13. Blood group 14. Hepatitis B & C 15. HTS 16. Pregnancy Test 17. LLITN 18. Physical examination 19. Para, MUAC 20. Pedal oedema 21. Breast examination 22. Vulva inspection 23. Obstetric history 24. Add breast feeding history 25. To include questions for TB 26. Replace village with place of residence on cover page 27. Colour of the health passport cover should be green 28. Centralized/certified publisher of the booklets 29. The content in the booklet to be ordered chronologically 30. Flow of pages 31. Personal information 32. Obstetric history 33. ANC consultation Record 34. Education & Counselling 35. Summary of Delivery 36. Increase the size of the booklet to accommodate the suggested columns especially on ANC consultation records e.g. HTS 37. Document EDD by Date & scan 38. HTSS done 3X and results incorporated on objective data section 39. Additional vital signs; temperature, pulse rate, respiratory rate 40. Have separate pages for history, examination and investigations 41. PT at first ANC visit 42. Fundal height documented in CM 43. For notebook size 5 use one page for objective and subjective information for each pregnancy-space for four pregnancies 44. If we maintain same size Health passport-separate pages for History, examination and investigation 45. TTV section shifted to middle page 46. Shedding of columns to specify timing of drugs administration and investigations??G2 47. Update ART section according to new guidelines 48. Space for ITN 49. Remove symphisiotomy because it is no longer practiced 50. Remove bony pelvic assessment because it is not practiced during Antenatal period & it can increase the chances of infection 51. Calcium should not be included, it cannot be given routinely but on special cases 52. Obstetric history add number of ceasarian section done 53. GA column: GA by LMP/USS 54. Syphillis, HIV & Hepatitis B should be on the same page 55. Two columns for EDD(LMP & USS) 56. Add table for Urinalysis 57. PMTCT: EMTCT 58. Syphilis & Hepatitis B should go to page 6 59. WHO HIV stage to be removed 60. CD4 count to be removed 61. Remove NVO, AZT & 3TC columns 62. Include food supplementation 63. Space for documenting ITN administration 64. Include a space or page for checklist of all client education/ counselling components | Management  -Control of procurement of tools  -Funding to process health passport books  -Sustainability  Implementation  -Human resources, e.g. skilled USS providers and lab technicians  -Still have old booklets  -Work load  -Lack of commitment  -Lack of training/orientations  -Women changing health passport for various reasons  -No electronic back up  -Attitude of health workers: deliberate omission o  Resources  -Lack of PT kits, urine test kits, and haemacue kits, FBC machines  -No provision of health passport by MoH  -Unavailability of calcium supplements  Implementation climate barriers  -Lack of waterproof cover | -Availability of trained midwives  -MoH has guidelines and training package  -Motivation to pregnant women  -Existence of partnership/developmental  -Availability of safe motherhood program  -Providers’ knowledge on use of tools  -Readily available equipment and supplies  -Political will  -Adequate planning, financing, and budgeting  -Community engagement  -Leadership and governance | Political  -MoH to resume publishing of health passport  -Politicians should adopt it (G3)  -Service charter  -Use of influential leaders  Economic  -Funding should be made available to print the tool  -DIP to put in plans in the procurement of resources  -DIP to plan on trainings use of new ANC  -Transparency and accountability  -Monitoring and evaluation  Sociological  -Should be a tool that will be accepted by the community (G3)  -Sensitisation and orientation of health workers  Technological  -Incorporate antenatal care into HMIS (G3)  -Introduce electronic data system  -Should have a bar code  -electronic specialized consultation using social medical platforms  Legal  -Use only printers acknowledged by RHD  -Policies to have legal backing  Environmental  -It should be environmental friendly  -Infrastructure |

**Additional File 4. Affinity diagram/reduction summary from whole group consensus discussion for Mangochi workshop**

| **Gaps Identified in the current tool (Women’s health passport book/ANC card)** | **Changes to be made to the women’s health passport book.** | **Barriers to implementation of the newly developed women’s health passport book.** | **Facilitators of implementation of the newly developed women’s health passport book** | **Strategies to optimally implement the new women’s health passport book** |
| --- | --- | --- | --- | --- |
| -Current ANC tool does not have space for USS results  -No drug history e.g., antihypertensives  -Nowhere to document Hep B and C status  -No mental health assessment  -No TB risk assessment  -No history of GBV/IPV  -No observations of MUAC and GAIT  -Inadequate space for documenting history of complaints  - No breastfeeding history  - No Lab investigations i.e., Sugar and Leucocytes, urine protein, leucocytes, acetone, nitrate, and pregnancy test  -No Space for documentation of drugs e.g., calcium  -No documentations of client education and counselling  -No surgical history including pelvic surgeries and myomectomy  -No space for vaginal inspection, bony pelvic assessment, and genital ulcers  -No space for general examination | -Add more pages   1. miscarriages 2. Hep b and Blood group 3. 3 middle pages   -Remove WHO clinical stage  -USS for dating on the middle  -Substitute HTC with ITN on the middle  -To include Albendazole at the middle  -On TTV, include 4 boxes and reminders of schedule  -USS  -A5 matrix with information for minimum of 3 pregnancies (to follow up all pregnancies in woman’s life time)  - Lab results comprehensive  - high risk highlighters  - FHR not fetal heart sounds | -Facility stockouts  -Lack of laboratory facilities  -Unavailability of reagents  -Printing rights not protected | -Presence of trained staff in USS (Medical Officers in districts and health centres)  -Buy in policy makers  -Good support form practitioners | -Dissemination of information on importance of documentation  -Centralization of health passport printing  -Education/sensitization  -Tally with national M & E framework |
