# Supplementary material for: Developing comprehensive woman hand-held case notes to improve quality of antenatal care in low-income settings: participatory approach with maternal health stakeholders in Malawi
Source: BMC Health Serv Res. 2024 May 15;24:628. doi: 10.1186/s12913-024-10922-3 (PMC11094996; doi:10.1186/s12913-024-10922-3)
Supplement: Supplementary file 2 — Supplementary Material 2 [file 12913_2024_10922_MOESM2_ESM.pdf]

HMIS-10 VersionXXXX June 2023

MALAWI HEALTH PASSPORT
WOMAN HEALTH PROFILE

NAME ______________________________

DATE OF BIRTH ______________________ Day / Month / Year

RESIDENTIAL AREA ______________________________

Please bring this book each time you come to see the nurse or doctor. If you are pregnant, remember to carry this book at all times during your pregnancy.

Issuing person: ______________________________

Issued date: ______________________________

Place of Issue: ______________________________

CONFIDENTIAL

NDONDOMEKO YA CHISAMALIRO CHAULEMU KWA AMAYI NDI MAKANDA PAUCHEMBERE[1]

1. Muli ndi ufulu otetezedwa ku nkhanza kapena kuvulazidwa pamene mukulandira thandizo la chipatala.

2. Muli ndi ufulu olandira uthenga, wovomereza kapena kukana thandizo limene achipatala anena kuti mulandire mutadziwitsidwa mokwanira, komanso zisankho zanu zilemekezedwe kuphatikizapo kusankha munthu okhala naye polandira kapena kukana thandizo panthawi yomwe muli m’chipatala.

3. Muli ndi ufulu othandizidwa mwachinsinsi komanso kusungilidwa chinsinsi.

4. Aliyense ndi munthu payekha kuchokela panthawi yobadwa ndipo muli ndi ufulu othandizidwa mwaulemu ndi molemekezedwa monga munthu.

5. Muli ndi ufulu olandira thandizo mofanana ndi mopanda tsankho.

6. Muli ndi ufulu olandira thandizo lachipatala ndikukhala ndi thanzi monga kuyenera.

7. Muli ndi ufulu wokhala momasuka, wochita chiganizo panokha ndi wosasungidwa m’chipatala mokakamizidwa.

8. Mwana wanu ali ndi ufulu okhala nanu ngati makolo ake kapena omuyang’anira.

9. Mwana wanu ali ndi ufulu odziwika ndi dzina komanso kukhala nzika ya dziko akangobadwa.

10. Muli ndi ufulu wa madyedwe oyenera ndi madzi aukhondo.

[1]*Contribution by the White Ribbon Alliance*

RESPECTFUL MATERNITY CARE CHARTER[1]
Universal rights of women and newborns

1. You have the right to freedom from harm and ill-treatment.

2. You have the right to information, informed consent and respect for your choices and preferences, including compassion of choice during maternity care and refusal of medical procedures.

3. You have the right to privacy and confidentiality.

4. From the moment of birth, you have the right to be treated with dignity and respect.

5. You have the right to equality, freedom from discrimination and equitable care.

6. You have the right to healthcare and to the highest attainable level of health.

7. You have the right to liberty, autonomy, self determination and freedom from arbitrary detention.

8. Your child has the right to be with you as his/her parents or guardians.

9. Your child has the right to an identity and nationality from birth.

10. You have the right to adequate nutrition and clean water.

[1] Contribution by the White Ribbon Alliance Malawi.

EDUCATIONAL CONTENT FOR PREGNANT WOMEN

Your pregnancy journey should be a positive experience.

There are key red flags which you should know about. If you have any of the problems below you should urgently seek medical attention from you nearest healthcare facility.

a) Severe abdominal pain: Abdominal discomfort is not uncommon during pregnancy, but if it becomes severe, does not go away with simple rest, or comes at the same time as vaginal bleeding then go straight to your nearest health facility.

b) Vaginal bleeding: Any vaginal blood loss should be reported immediately to your nearest health facility.

c) Water leaking from the vagina: Your waters may break before labour starts at any time during pregnancy. If you have watery loss from your vagina, which you can't control, report to your nearest health facility, immediately.

d) Abnormal vaginal discharge: It is normal to have increased vaginal discharge when pregnant. This should be clear white and not unpleasant. If there is a colour change, offensive smell, or you feel sore or itchy, visit the nearest health facility.

e) Infections: High temperatures above 38oC, fever and chills, pain or frequently passing urine, diarrhoea and vomiting, rash, sore throat or respiratory infection, painful red blisters or sores around the vagina or bottom or thighs should all prompt you to visit the nearest health facility.

f) Severe chest pain or breathless or you notice an increase in your heart rate.

g) Severe headaches, blurred vision or spots before your eyes, obvious swelling of hands and face and severe pain below your ribs and/or vomiting.

h) Feeling persistently depressed or anxious, or having thoughts of harming yourself or your baby.

i) Baby movements: Once you have started feeling your baby move, you should feel these movements regularly each day. Any change in movements needs checking at your nearest health facility.

It is very important for you to attend antenatal visits throughout your pregnancy journey. It is required that you have 8 contacts with a healthcare provider. At each visit, checks will be done and advice will be given to you depending on the findings and stage of your pregnancy.

Aim to stay in the best mental health state as possible, seek support if you need it.

Report any issues of Gender-Based Violence.

Ensure that you are eating healthily, including taking Folic acid from the point when you are planning to become pregnant, and take iron tablets and multi-vitamins during your pregnancy.

Always sleep under an insecticide treated bednet and have preventative treatment for Malaria during your pregnancy.

Ensure you have all the recommended vaccines from your health care provider.

Avoid smoking or drinking alcohol.

Avoid any helbal remedies, and seek medical advice before taking any medications.

You are strongly advised to come with your partner to the ANC clinic during your pregnancy

REGISTRATION *(to be filled by the doctor or nurse on each visit)*

Date of Visit	Facility ID	Registration No.	Purpose of Visit	Details

GENERAL HISTORY (tick, circle or fill as appropriate)

A. Personal Details

Marital Status: Single ☐ Married ☐ Separated ☐ Divorced ☐ Widowed ☐

Occupation: ______________________________

Religion/Faith: ______________________________

Smoker: Yes ☐ No ☐ Used Tobacco in previous 12 months? Yes ☐ No ☐

Alcohol use: Yes ☐ No ☐

Use of other drugs and substances Yes ☐ No ☐

Blood Group: ____________________

Any physical/sensory disability: ____________________

Comments

B. Family History (The term 'family' here means blood relatives only e.g. your children, your parents, grandparents, brothers and sisters, uncles and aunts and their children i.e. first cousins)

Has anyone in your family had:

	No	Yes		No	Yes
Allergies	☐	☐	Stillbirths or multiple miscarriages	☐	☐
Mental illness	☐	☐	Sudden infant death	☐	☐
Neoplasms	☐	☐	Hearing loss from childhood	☐	☐
Asthma	☐	☐	Heart problems from birth	☐	☐
Diabetes	☐	☐	Abnormalities present at birth	☐	☐
Cardiovascular diseases	☐	☐	Inherited metabolic disorder	☐	☐
Endocrine diseases	☐	☐	Comments:		
Hypertension	☐	☐			
Rheumatism	☐	☐			
Sickle cell disease	☐	☐			
Epilepsy	☐	☐			
Other diseases	☐	☐			
-					
-					

C. Past Medical, Surgical and Vaccination History

I. Medical History

Do you have/have you had?

Condition	No	Yes	Date of Onset (dd.mm.yyyy)	Details (e.g. medication, type of condition)
Arthritis	☐	☐	______	______
Asthma	☐	☐	______	______
Depression/Anxiety	☐	☐	______	______
Other Mental Illness	☐	☐	______	______
Diabetes	☐	☐	______	______
Epilepsy	☐	☐	______	______
Haemophilia	☐	☐	______	______
Heart attack	☐	☐	______	______
Hypertension	☐	☐	______	______
Jaundice	☐	☐	______	______
Mental illness	☐	☐	______	______
PID[2]	☐	☐	______	______
Pneumonia	☐	☐	______	______
Rheumatism	☐	☐	______	______
Sickle cell	☐	☐	______	______
STI[3]	☐	☐	______	______
Tuberculosis	☐	☐	______	______

Others:
(specify)

---

[2] *Pelvic Inflammatory Disease*
[3] *Sexually Transmitted Infection*

II. Surgical History

**Diagnosis**	**Date of surgery** (dd.mm.yyyy)	**Type of surgery performed**	**Comments/Details**

III. **Immunisation** *(Healthcare provider should remind the client of dates to visit the facility)*

Tetanus Toxoid Immunisation

Dose	Date administered (dd.mm.yyyy)	Initials/signature of staff	Schedule
TTV/TD1			Start
TTV/TD2			4 weeks
TTV/TD3			6 months
TTV/TD4			1 year
TTV/TD5			1 year later

Other Vaccines (e.g. COVID-19 vaccine etc)

Dose	Date administered (dd.mm.yyyy)	Initials/signature of staff

FAMILY PLANNING
EXAMINATION

1	Breastfeeding a baby less than 8 months old		
2	Blood Pressure more than 150/100		
3	Skin or eyes appear yellowish		
4	Lump in breast		
5	Swollen or throbbing varices		
6	Heavy or frequent menses		
7	Difficult breathing		
8	Frequent headache		
9	Sign of pregnancy		
10	Taking Rifampicine or Grisiofulvin		
11	Sign of inflammation		
12	Tumor		
13	Vaginal discharge		

Method Selected (tick method selected)

Condom	
Lo-femenal	
Ovrette	
Depo IM	
Depo SC (Sayana Press)	
Norplant	
IUCD	
Male Sterilisation	
Female Sterilisation	

Selection Criteria

1. Lo-female; only if 1-9 are No
2. Depo IM, Depo SC, Ovrette, Norplant; only if 2-6 and 9 are No
3. IUCD only if 11-13 are No

Date Visited	BP/Weight/Complaints/advice	Supply given	Date of next appointment

HUMAN IMMUNODEFICIENCY VIRUS (HIV) TESTING AND COUNSELLING

HIV affects the body's ability to fight infection. When detected and treated fully, you can live a healthy life, and there is minimal risk to pass the infection to the baby. A negative test 'now' does not mean youare negative. Every pregnant woman has to be tested for HIV and be offered counselling during their antenatal care, till the time of birth.

Date	Facility name	HTC Serial No.	Outcome 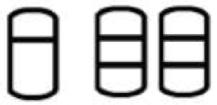	Action taken/recommenda-tions	CD4 Count (If applicable)	Viral load (If applicable)	Provider Sign

Comments:

Date________________ **Diagnosis/Lab/Treatment/Notes**

Pregnancy 2

ANTENATAL CONSULTATION RECORD

Care provider should ensure that all concerns of the woman are discussed thoroughly. In addition, all important pregnancy symptoms should be reiterated in a satisfactory manner.

LNMP-Last Normal Menstrual Period; EDD-Estimated Date of Delivery; USS-Ultrasound scan

ANC facility name	Registration Number	ANC register page	Age	Gravida	Para	Miscarriage	LNMP	EDD-By Date -By USS	Singleton ☐ Multiple pregnancy ☐

Demographics

Education level	
Religion	
Occupation	
Marital status	

Past Obstetric History

Circle where it applies and provide details

Deliveries	1	2	3	4	**5***
Miscarriage	0	1	**2***	3	
Stillbirths	0	1	**2***		
C/Section	0	1	**2***		
Vacuum Extraction	**Y***	N			
APH	**Y***	N			
PPH	**Y***	N			
Multiple gestation	**Y***	N			
Pre-eclampsia	**Y***	N			
Fistula repair	**Y***	N			
Past breastfeeding problem	**Y***	N			
Mental illness in pregnancy/postpar-tum	**Y***	N			

*** = referral to high risk care facility**

Comments

Medical History

Circle where it applies and provide details

Asthma	Y	N
Hypertension	Y	N
Diabetes	Y	N
If Yes, which type: Type I☐ Type II☐		
Epilepsy	Y	N
Renal disease	Y	N
Mental illness (bipolar disorder, depression, anxiety, psychosis)	Y	N
Heart disease	Y	N
Other diseases	Y	N

Comments:

Client with medical conditions require referral to high risk care facility (district/central hospital)

TB Risk Assessment

Fever	Y	N
Cough	Y	N
Weight Loss	Y	N

TB test result:
Pos ☐ **Neg** ☐

Treatment (if positive):

A cough lasting longer than 2 weeks, unexplained weight loss, nights sweats/fever and loss of appetite warrants a TB test

Booking Investigation

Date	Test Result	Taken	Result
	HIV 1 (At first contact/visit)		
	Hb 1 (At first contact/visit)		
	Syphilis		
	Blood Group		
	Hepatitis B		
	Pregnancy Test		
	Blood Sugar		
	Urine Nitrites		
	Urine Leucocytes		
	Urine Protein		
	Urine Sugar		
**Third trimester investigation**			
	HIV 2		
	Hb 2		

Physical Examination

Height	150cm +	<150cm

Vaginal Examination

Not Done ☐

Examination explained and permission obtained.☐
(Tick if permission obtained and there is need to do the examination)

Vulva and Vagina____________________

Surgical/Gynae History

Myomectomy	Y	N	
Ectopic	Y	N	
Others:	Y	N	

Screened for Cervical Cancer before?
Yes ☐ No ☐
(If Yes)
Pap smear? ____________
Date____________
Result____________

VIA? ________________
Date____________
Result____________

If positive, what action was taken?
____
____

Counselling

Health Talks	Date Given 1	Date Given 2
**Your Pregnancy**		
Parental preparedness		
Nutrition		
Danger signs		
Fetal movements		
Mental health		
Domestic/gender based violence		
HIV		
Male involvement in ANC		
Smoking/tobacco use		
Alcohol/substances use		

Counselling

Health Talks	Date Given 1	Date Given 2
**Your Birth**		
Labour and birth preparedness		
Birth companion		
**After Birth**		
Breastfeeding and complementary feeding		
Breast care		
Contraception		

Insecticide Treated Net given (Yes ☐ No☐ (All pregnant women should be given an Insecticide Treated Net)

DELIVERY PLAN

Planned Delivery Place______________________ Plan for Transport_________________________ Birth companion discussed? **Yes** ☐ **No** ☐

(Provider should discuss with client on birth companion and options available)

Whom would you want to have as your companion? ____

Fill in information for every pregnancy/visit
Gest = Gestation Age; SFH = Symphysis Fundal Height; Pres=Presentation; MUAC=Mid-upper Arm Circumference; BP=Blood Pressure; SP=Sulfadoxine-pyrimethamine; FeFol=Iron and Folic Acid; Alb=Albendazole; NVP=Nevirapine; ART=Antiretroviral therapy; CPT=Cotrimoxazole Preventive Therapy

Visits	Visit Date	Gest (Wks + Days)	SFH (cm)	Pres	Fetal movements		Fetal Heart	Pallor* (Y/N)	Weight (kg)	MUAC (cm)	BP* (mmHg)	Urine protein*	Mental Wellbeing Check	Medication/Preventive Measures (Dose given)						Next visit date	Sign
					Felt	Dis-cussed								SP*	FeFol	Alb	Calcium with Vitamin D3	Baby NVP	On ART+CPT (Y/N/NA)		
1																					
2																					
3																					
4																					
5																					
6																					
7																					
8																					

SP*: A minimum of 3 doses should be given across the period. If not given SP, specify drug given here: _______________
BP*: Systolic BP of 140mmHg or more, refer client accordingly. Systolic BP of 160mmHg or more, urgent treatment and referral. Urine protein*: 2+ with hypertension, refer client. Pallor *: Y means present and N means absent

ULTRASOUND SCAN DETAILS

The World Health Organisation (WHO) endorses the use of one ultrasound scan before 24 weeks gestation for all pregnant women.
Where opportunity allows for the woman to have a print-out of the scan, the care provider should give one.

FH-Fetal Heart; BPD-Bi-parietal diameter; HC-Head circumference; AC-Abdominal circumference; FL-Femur Length; CRL-Crown Rump Length; GA-Gestational Age; FW-Fetal Weight; Pres-Presentation/Lie; Liquor volume (indicate if LOW, ADEQUATE, or HIGH); EDD-Estimated Date of Delivery.

Date	Trimester	Facility Name	No. of fetuses	FH rate	BPD	HC	AC	FL	CRL	GA	Estimated FW	Pres/ lie	Placental location	Liquor Volume	EDD	Scanner sign
Comments:																
Comments:																
Comments:																

International Symphysis-Fundal Height Standards

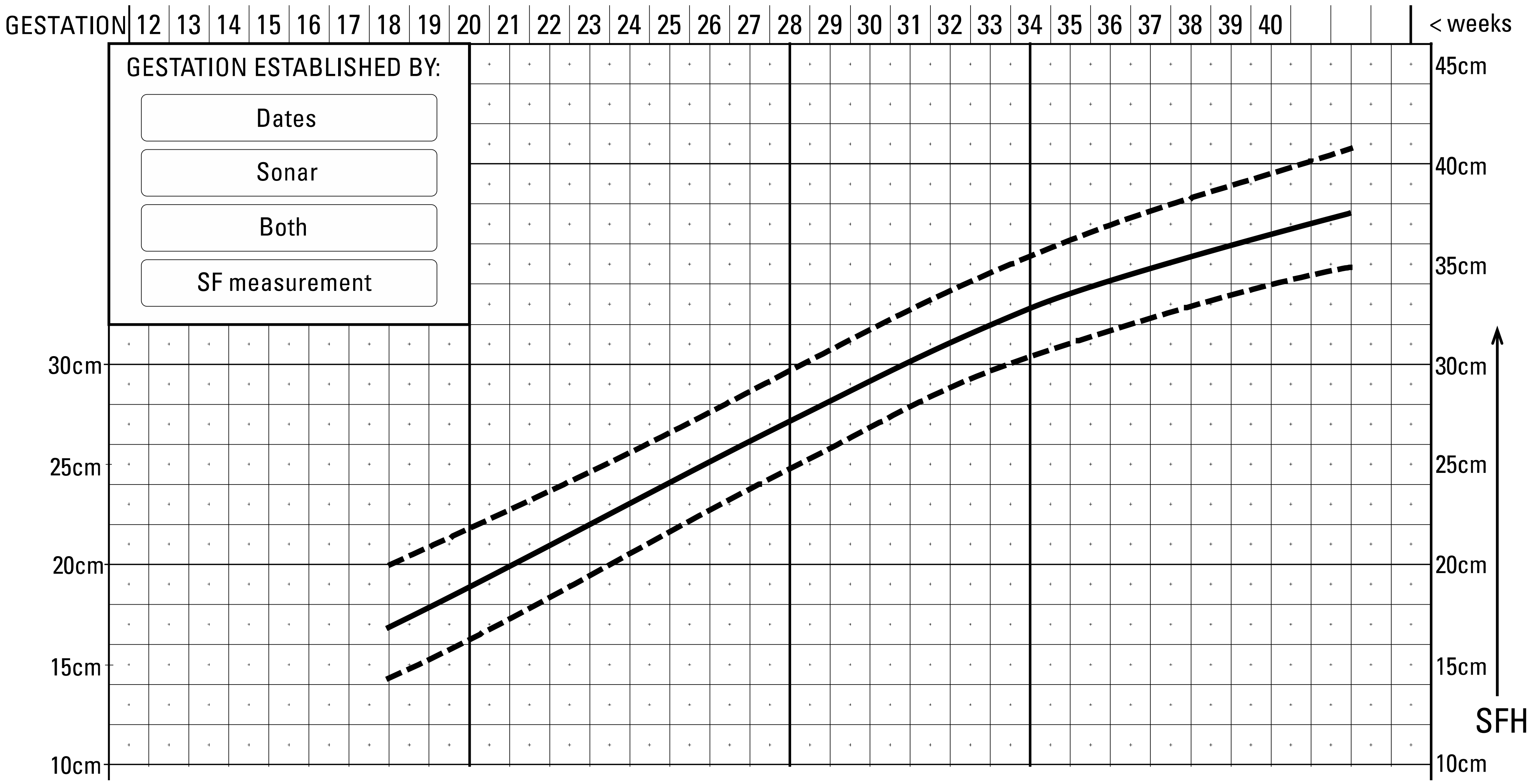

DELIVERY SUMMARY

Date of Delivery___________________________

Time______________________________________

Place of Delivery: (tick)

Central Hospital ☐	District or CHAM Hospital ☐	Health Centre ☐	Home ☐	Private Hospital ☐

Type of Delivery: ____

Partograph or labour care guide used? Yes/No

Date of Discharge: ________________________________

Birthweight: ___________________ Sex: _________________

OPV 0 given and recorded? Yes ☐ No ☐

BCG Given and recorded? Yes ☐ No ☐

Discharge weight: ___________________________________

Child Health Passport Issued? Yes ☐ No ☐

(if not, make effort to help the mother get a Child's Health passport)

Comment on overall condition of the infant:

……

……

……

Checklist:

Care provider should ensure the following are discussed (tick as appropriate)

How to exclusively breastfeed	
Advantages of exclusive breastfeeding	
Position and attachment of baby when feeding	
Expressing, storage and feeding of breastmilk	
Contraception information	
Contraception advice	
Mental wellbeing	

POST NATAL CHECK

Date	Weight	BP	Hb	Breastfeeding progress/comment	Comment on any other complaints, assessments

Date________________ **Diagnosis/Lab/Treatment/Notes**

ANTENATAL CONSULTATION RECORD

Care provider should ensure that all concerns of the woman are discussed thoroughly. In addition, all important pregnancy symptoms should be reiterated in a satisfactory manner.

LNMP-Last Normal Menstrual Period; EDD-Estimated Date of Delivery; USS-Ultrasound scan

ANC facility name	Registration Number	ANC register page	Age	Gravida	Para	Miscarriage	LNMP	EDD-By Date<br>-By USS	Singleton ☐<br>Multiple pregnancy ☐

Demographics

Education level	
Religion	
Occupation	
Marital status	

Past Obstetric History

Circle where it applies and provide details

Deliveries	1	2	3	4	**5***
Miscarriage	0	1	**2***	3	
Stillbirths	0	1	**2***		
C/Section	0	1	**2***		
Vacuum Extraction	**Y***	N			
APH	**Y***	N			
PPH	**Y***	N			
Multiple gestation	**Y***	N			
Pre-eclampsia	**Y***	N			
Fistula repair	**Y***	N			
Past breastfeeding problem	**Y***	N			
Mental illness in pregnancy/postpar-tum	**Y***	N			

*** = referral to high risk care facility**

Comments

Medical History

Circle where it applies and provide details

Asthma	Y	N
Hypertension	Y	N
Diabetes	Y	N
If Yes, which type: Type I☐ Type II☐		
Epilepsy	Y	N
Renal disease	Y	N
Mental illness (bipolar disorder, depression, anxiety, psychosis)	Y	N
Heart disease	Y	N
Other diseases	Y	N

Comments:

Client with medical conditions require referral to high risk care facility (district/central hospital)

TB Risk Assessment

Fever	Y	N
Cough	Y	N
Weight Loss	Y	N

TB test result:
Pos ☐ **Neg** ☐

Treatment (if positive):

A cough lasting longer than 2 weeks, unexplained weight loss, nights sweats/fever and loss of appetite warrants a TB test

Booking Investigation

Date	Test Result	Taken	Result
	HIV 1 (At first contact/visit)		
	Hb 1 (At first contact/visit)		
	Syphilis		
	Blood Group		
	Hepatitis B		
	Pregnancy Test		
	Blood Sugar		
	Urine Nitrites		
	Urine Leucocytes		
	Urine Protein		
	Urine Sugar		
**Third trimester investigation**			
	HIV 2		
	Hb 2		

Physical Examination

Height	150cm +	<150cm

Vaginal Examination

Not Done ☐

Examination explained and permission obtained.☐
(Tick if permission obtained and there is need to do the examination)

Vulva and Vagina____________________

Surgical/Gynae History

Myomectomy	Y	N	
Ectopic	Y	N	
Others:	Y	N	

Screened for Cervical Cancer before?
Yes ☐ No ☐
(If Yes)
Pap smear? ____________
Date____________
Result____________

VIA? ____________________
Date____________
Result____________

If positive, what action was taken?
____
____

Counselling

Health Talks	Date Given 1	Date Given 2
**Your Pregnancy**		
Parental preparedness		
Nutrition		
Danger signs		
Fetal movements		
Mental health		
Domestic/gender based violence		
HIV		
Male involvement in ANC		
Smoking/tobacco use		
Alcohol/substances use		

Counselling

Health Talks	Date Given 1	Date Given 2
**Your Birth**		
Labour and birth preparedness		
Birth companion		
**After Birth**		
Breastfeeding and complementary feeding		
Breast care		
Contraception		

Insecticide Treated Net given (Yes ☐ No☐ (All pregnant women should be given an Insecticide Treated Net)

DELIVERY PLAN

Planned Delivery Place______________________ Plan for Transport___________________________ Birth companion discussed? **Yes** ☐ **No** ☐

(Provider should discuss with client on birth companion and options available)

Whom would you want to have as your companion? ____

Fill in information for every pregnancy/visit
Gest = Gestation Age; SFH = Symphysis Fundal Height; Pres=Presentation; MUAC=Mid-upper Arm Circumference; BP=Blood Pressure; SP=Sulfadoxine-pyrimethamine; FeFol=Iron and Folic Acid; Alb=Albendazole; NVP=Nevirapine; ART=Antiretroviral therapy; CPT=Cotrimoxazole Preventive Therapy

Visits	Visit Date	Gest (Wks + Days)	SFH (cm)	Pres	Fetal movements		Fetal Heart	Pallor* (Y/N)	Weight (kg)	MUAC (cm)	BP* (mmHg)	Urine protein*	Mental Wellbeing Check	Medication/Preventive Measures (Dose given)						Next visit date	Sign
					Felt	Dis-cussed								SP*	FeFol	Alb	Calcium with Vitamin D3	Baby NVP	On ART+CPT (Y/N/NA)		
1																					
2																					
3																					
4																					
5																					
6																					
7																					
8																					

SP*: A minimum of 3 doses should be given across the period. If not given SP, specify drug given here: _________________
BP*: Systolic BP of 140mmHg or more, refer client accordingly. Systolic BP of 160mmHg or more, urgent treatment and referral. Urine protein*: 2+ with hypertension, refer client. Pallor *: Y means present and N means absent

ULTRASOUND SCAN DETAILS

The World Health Organisation (WHO) endorses the use of one ultrasound scan before 24 weeks gestation for all pregnant women.
Where opportunity allows for the woman to have a print-out of the scan, the care provider should give one.

FH-Fetal Heart; BPD-Bi-parietal diameter; HC-Head circumference; AC-Abdominal circumference; FL-Femur Length; CRL-Crown Rump Length; GA-Gestational Age; FW-Fetal Weight; Pres-Presentation/Lie; Liquor volume (indicate if LOW, ADEQUATE, or HIGH); EDD-Estimated Date of Delivery.

Date	Trimester	Facility Name	No. of fetuses	FH rate	BPD	HC	AC	FL	CRL	GA	Estimated FW	Pres/ lie	Placental location	Liquor Volume	EDD	Scanner sign
Comments:																
Comments:																
Comments:																

International Symphysis-Fundal Height Standards

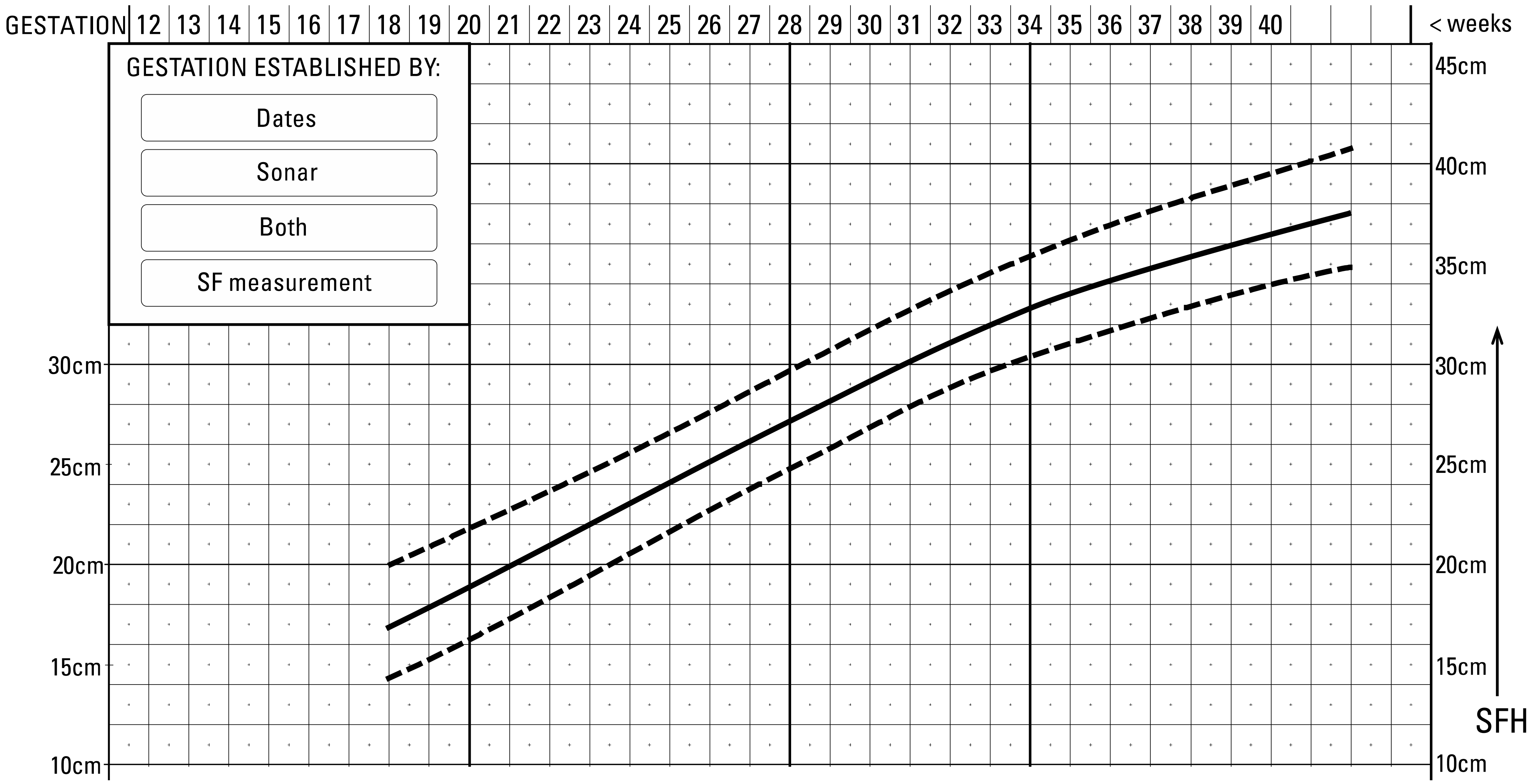

DELIVERY SUMMARY

Date of Delivery__________________________

Time____________________________________

Place of Delivery: (tick)

Central Hospital ☐	District or CHAM Hospital ☐	Health Centre ☐	Home ☐	Private Hospital ☐

Type of Delivery: ____

Partograph or labour care guide used? Yes/No

Date of Discharge: ________________________________

Birthweight: __________________ Sex: ________________

OPV 0 given and recorded? Yes ☐ No ☐

BCG Given and recorded? Yes ☐ No ☐

Discharge weight: __________________________________

Child Health Passport Issued? Yes ☐ No ☐

(if not, make effort to help the mother get a Child's Health passport)

Comment on overall condition of the infant:

....

....

....

Checklist:
Care provider should ensure the following are discussed (tick as appropriate)

How to exclusively breastfeed	
Advantages of exclusive breastfeeding	
Position and attachment of baby when feeding	
Expressing, storage and feeding of breastmilk	
Contraception information	
Contraception advice	
Mental wellbeing	

POST NATAL CHECK

Date	Weight	BP	Hb	Breastfeeding progress/comment	Comment on any other complaints, assessments

Date________________ **Diagnosis/Lab/Treatment/Notes**

Date________________ Diagnosis/Lab/Treatment/Notes

Date________________ **Diagnosis/Lab/Treatment/Notes**

Date_________________ **Diagnosis/Lab/Treatment/Notes**

Date________________ Diagnosis/Lab/Treatment/Notes

Date________________ Diagnosis/Lab/Treatment/Notes

Date________________ Diagnosis/Lab/Treatment/Notes

Date________________ **Diagnosis/Lab/Treatment/Notes**

Pregnancy 1

ANTENATAL CONSULTATION RECORD

Care provider should ensure that all concerns of the woman are discussed thoroughly. In addition, all important pregnancy symptoms should be reiterated in a satisfactory manner.

LNMP-Last Normal Menstrual Period; EDD-Estimated Date of Delivery; USS-Ultrasound scan

ANC facility name	Registration Number	ANC register page	Age	Gravida	Para	Miscarriage	LNMP	EDD-By Date<br>-By USS	Singleton ☐<br>Multiple pregnancy ☐

Demographics

Education level	
Religion	
Occupation	
Marital status	

Past Obstetric History

Circle where it applies and provide details

Deliveries	1	2	3	4	**5***
Miscarriage	0	1	**2***	3	
Stillbirths	0	1	**2***		
C/Section	0	1	**2***		
Vacuum Extraction	**Y***	N			
APH	**Y***	N			
PPH	**Y***	N			
Multiple gestation	**Y***	N			
Pre-eclampsia	**Y***	N			
Fistula repair	**Y***	N			
Past breastfeeding problem	**Y***	N			
Mental illness in pregnancy/postpar-tum	**Y***	N			

*** = referral to high risk care facility**

Comments

Medical History

Circle where it applies and provide details

Asthma	Y	N
Hypertension	Y	N
Diabetes	Y	N
If Yes, which type: Type I☐ Type II☐		
Epilepsy	Y	N
Renal disease	Y	N
Mental illness (bipolar disorder, depression, anxiety, psychosis)	Y	N
Heart disease	Y	N
Other diseases	Y	N

Comments:

Client with medical conditions require referral to high risk care facility (district/central hospital)

TB Risk Assessment

Fever	Y	N
Cough	Y	N
Weight Loss	Y	N

TB test result:
Pos ☐ **Neg** ☐

Treatment (if positive):

A cough lasting longer than 2 weeks, unexplained weight loss, nights sweats/fever and loss of appetite warrants a TB test

Booking Investigation

Date	Test Result	Taken	Result
	HIV 1 (At first contact/visit)		
	Hb 1 (At first contact/visit)		
	Syphilis		
	Blood Group		
	Hepatitis B		
	Pregnancy Test		
	Blood Sugar		
	Urine Nitrites		
	Urine Leucocytes		
	Urine Protein		
	Urine Sugar		
**Third trimester investigation**			
	HIV 2		
	Hb 2		

Physical Examination

Height	150cm +	<150cm

Vaginal Examination

Not Done ☐

Examination explained and permission obtained.☐
(Tick if permission obtained and there is need to do the examination)

Vulva and Vagina____________________

Surgical/Gynae History

Myomectomy	Y	N	
Ectopic	Y	N	
Others:	Y	N	

Screened for Cervical Cancer before?
Yes ☐ No ☐
(If Yes)
Pap smear? ____________
Date____________
Result____________

VIA? __________________
Date____________
Result____________

If positive, what action was taken?
____
____

Counselling

Health Talks	Date Given 1	Date Given 2
**Your Pregnancy**		
Parental preparedness		
Nutrition		
Danger signs		
Fetal movements		
Mental health		
Domestic/gender based violence		
HIV		
Male involvement in ANC		
Smoking/tobacco use		
Alcohol/substances use		

Counselling

Health Talks	Date Given 1	Date Given 2
**Your Birth**		
Labour and birth preparedness		
Birth companion		
**After Birth**		
Breastfeeding and complementary feeding		
Breast care		
Contraception		

Insecticide Treated Net given (Yes ☐ No☐ (All pregnant women should be given an Insecticide Treated Net)

DELIVERY PLAN

Planned Delivery Place________________________ Plan for Transport_____________________________ Birth companion discussed? **Yes** ☐ **No** ☐

(Provider should discuss with client on birth companion and options available)

Whom would you want to have as your companion? ____

Fill in information for every pregnancy/visit
Gest = Gestation Age; SFH = Symphysis Fundal Height; Pres=Presentation; MUAC=Mid-upper Arm Circumference; BP=Blood Pressure; SP=Sulfadoxine-pyrimethamine; FeFol=Iron and Folic Acid; Alb=Albendazole; NVP=Nevirapine; ART=Antiretroviral therapy; CPT=Cotrimoxazole Preventive Therapy

Visits	Visit Date	Gest (Wks + Days)	SFH (cm)	Pres	Fetal movements		Fetal Heart	Pallor* (Y/N)	Weight (kg)	MUAC (cm)	BP* (mmHg)	Urine protein*	Mental Wellbeing Check	Medication/Preventive Measures (Dose given)						Next visit date	Sign
					Felt	Dis-cussed								SP*	FeFol	Alb	Calcium with Vitamin D3	Baby NVP	On ART+CPT (Y/N/NA)		
1																					
2																					
3																					
4																					
5																					
6																					
7																					
8																					

SP*: A minimum of 3 doses should be given across the period. If not given SP, specify drug given here: _________________
BP*: Systolic BP of 140mmHg or more, refer client accordingly. Systolic BP of 160mmHg or more, urgent treatment and referral. Urine protein*: 2+ with hypertension, refer client. Pallor *: Y means present and N means absent

ULTRASOUND SCAN DETAILS

The World Health Organisation (WHO) endorses the use of one ultrasound scan before 24 weeks gestation for all pregnant women.
Where opportunity allows for the woman to have a print-out of the scan, the care provider should give one.

FH-Fetal Heart; BPD-Bi-parietal diameter; HC-Head circumference; AC-Abdominal circumference; FL-Femur Length; CRL-Crown Rump Length; GA-Gestational Age; FW-Fetal Weight; Pres-Presentation/Lie; Liquor volume (indicate if LOW, ADEQUATE, or HIGH); EDD-Estimated Date of Delivery.

Date	Trimester	Facility Name	No. of fetuses	FH rate	BPD	HC	AC	FL	CRL	GA	Estimated FW	Pres/ lie	Placental location	Liquor Volume	EDD	Scanner sign
Comments:																
Comments:																
Comments:																

International Symphysis-Fundal Height Standards

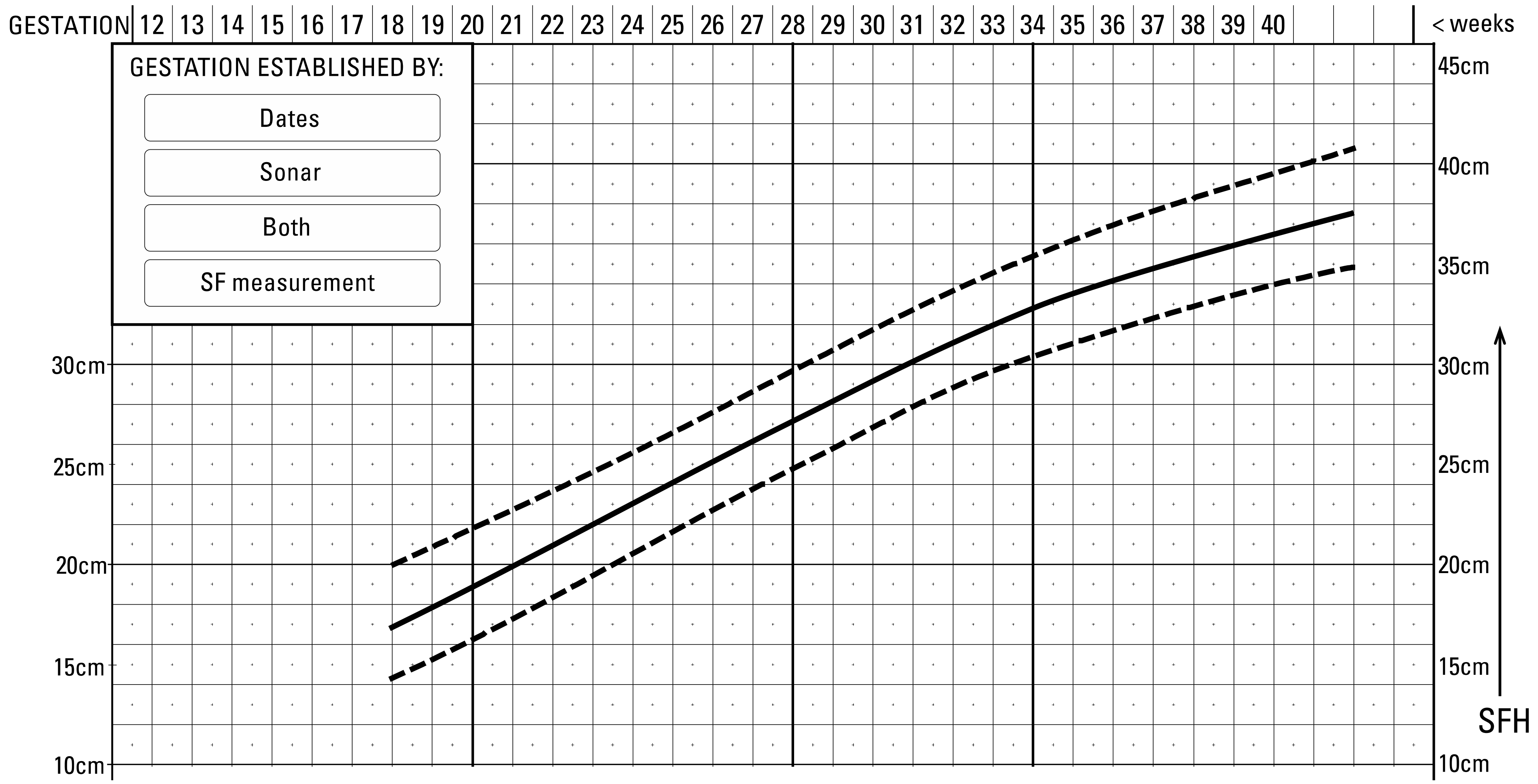

DELIVERY SUMMARY

Date of Delivery___________________________

Time______________________________________

Place of Delivery: (tick)

Central Hospital ☐	District or CHAM Hospital ☐	Health Centre ☐	Home ☐	Private Hospital ☐

Type of Delivery: ____

Partograph or labour care guide used? Yes/No

Date of Discharge: ___________________________________

Birthweight: ____________________ Sex: __________________

OPV 0 given and recorded? Yes ☐ No ☐

BCG Given and recorded? Yes ☐ No ☐

Discharge weight: _______________________________________

Child Health Passport Issued? Yes ☐ No ☐

(if not, make effort to help the mother get a Child's Health passport)

Comment on overall condition of the infant:

....

....

....

Checklist:

Care provider should ensure the following are discussed (tick as appropriate)

How to exclusively breastfeed	
Advantages of exclusive breastfeeding	
Position and attachment of baby when feeding	
Expressing, storage and feeding of breastmilk	
Contraception information	
Contraception advice	
Mental wellbeing	

POST NATAL CHECK

Date	Weight	BP	Hb	Breastfeeding progress/comment	Comment on any other complaints, assessments

Date________________ Diagnosis/Lab/Treatment/Notes

Date_________________ Diagnosis/Lab/Treatment/Notes

Date________________ Diagnosis/Lab/Treatment/Notes

Date________________ Diagnosis/Lab/Treatment/Notes

Date________________ Diagnosis/Lab/Treatment/Notes

Date________________ Diagnosis/Lab/Treatment/Notes

Date________________ **Diagnosis/Lab/Treatment/Notes**

Date________________ Diagnosis/Lab/Treatment/Notes

Date________________ Diagnosis/Lab/Treatment/Notes

Date________________ Diagnosis/Lab/Treatment/Notes

Date________________ **Diagnosis/Lab/Treatment/Notes**

Date________________ **Diagnosis/Lab/Treatment/Notes**

Date________________ Diagnosis/Lab/Treatment/Notes

Date________________ Diagnosis/Lab/Treatment/Notes

Date________________ **Diagnosis/Lab/Treatment/Notes**

Date________________ Diagnosis/Lab/Treatment/Notes

Date________________ Diagnosis/Lab/Treatment/Notes

Date________________ Diagnosis/Lab/Treatment/Notes

Date_________________ Diagnosis/Lab/Treatment/Notes

Date________________ Diagnosis/Lab/Treatment/Notes

REMINDER TO THE HEALTH PASSPORT HOLDER

1. This passport contains valuable information for your life.

2. This passport is issued to a female child at puberty or after finishing the use of child health passport whichever comes first.

3. If you had a child health passport, always keep that passport together with this one. The child health passport contains valuable information about your life, which helps your doctors and nurses to assess your problems correctly and give you the right treatment.

4. Don’t forget to carry this passport wherever you go for any consultation or any service.

5. This passport contains sections for: general history, family planning services, antenatal consultations and delivery for three pregnancies, and open space for general consultations, diagnosis and treatment.

6. You may be able to assess your health condition by looking into the circled diseases or conditions in the history section of this passport. Prior health conditions or other risk factors mean you need to be more careful about your health. Please ask your healthcare provider if you have any questions.

7. Keep this passport in a plastic pocket. You may use a clean empty packet of a 1-kg sugar bag to keep this passport to prevent it from getting worn-out.

CHONDE KUMBUKILANI IZI

1. Kabuku ka kali ndi mbiri yofunika kwambiri ya moyo wanu.

2. Kabuku ka kamapelekedwa kwa mtsikana aliyense akatha msikhu, kapena pomwe kabukhu ka ku umwana kadzadza kapena kutha.

3. Sungani pamodzi kabuku aka ndi kabuku kamene kamaperekedwa munthu akangobadwa ngati munali nako. Kabuku kamene kamaperekedwa munthu akangobadwa kamakhala ndi mbiri yofunikira ya moyo imene imathandiza ma dotolo ndi anamwino kuti afufuze vuto lanu ndi kukuthandizani moy-enera.

4. Onetsetsani kuti mwatenga kabu aka popita kwina kuli konse komwe mukukafuna thandizo la za umoyo.

5. Ka buku aka kali ndi magawo awa: mbiri yanu, za kulera, za ku sikelo ya amayi oyembekezera ndi uchembere, ndi gawo la thandizo lina lililonse lomwe mwalandira lokhudza umoyo wanu.

6. Mukhoza kuona ndi kudziwa za umoyo wanu poona matenda amene azunguzidwa mu mbali ya mbiri yanu mukabukuka. Mabvuto a za umoyo wanu a m'mbuyo ndi zina zokuyiyikani pa chiopsyezo zikutanthauza kuti mukuyenera kusamalitsa ndi umoyo wanu. Ngati muli ndi funso afunseni a zaumoyo kapena a dotolo/namwino amene ali m'dera lanu kapena kuchipatala cha pafupi.

7. Sungani kabukuka mu pepala la plastic, monga pepala la sugar loti wathamo, kuti kasanyowe ndi kung'ambika

MAPHUNZIRO KWA AMAYI OYEMBEKEZERA

Kukhala oyembekezera sichiphinjo, ndipo ulendo wanu umayenela ukhale wa myaaaaa.
Pali zizindikiro zoopsa zomwe mukuyenera kuzidziwa. Ngati muli ndi ena mwa mavuto omwe ali mmusiwa mukuyenera kupeza chithandizo mwansanga ku chipatala chomwe muli nacho pafupi.

a). Kupweteka kwambiri kwa m'mimba: Nthawi yomwe muli ndi pakati, m'mimba mumapweteka ndithu, koma ngati kwapitiliza muyeso, simukusiya ngakhale mutazipumitsa, kapena mukutayanso magazi panthawi yomweyo, pitani kuchipatala chomwe muli nacho pafupi.

b). Kutaya Magazi: Ngati mukutaya magazi kuchokela ku malo obisika, chonde adziwitseni a zaumoyo kuchipatala chomwe muli nacho pafupi.

c) Madzi kutayika kuchokera ku malo obisika: nsengwa imatha kusweka matenda asanayambe nthawi ina iliyonse pamene munthu ali ndi mimba. Ngati mukutaya madzi kuchokera Kumalo obisika, omwe panokha simungawaimitse, pitani kuchipatala chomwe muli nacho pafupi nthawi yomweyo.

d). Chikazi chosakhala bwino: Chikazi chochuluka chimatuluka pamene munthu ali ndi mimba. Chikazi chimayenera kukhala choyera komanso chosanukha. Ngati chasintha mtundu, kununkha, kapena mukumva kupweteka ngakhale kuyabwa, pitani kuchipatala chomwe muli nacho pafupi.

e) Nthenda: Kutentha kwa thupi kopitilira ma digili 38, kunjenjemera, kupweteka kwa m'thupi kapena kukodza pafupipafupi, kutsegula m'mimba ndi kusanza, tizilonda, nsungu za pakhosi kapena chimfine ndi chifuwa, matuza ofiira opweteka kapena mabala ku malo obisika kapena m'matako ngakhale mu ntchafu, zikuyenera kukupangitsani kupita kuchipatala chomwe muli nacho pafupi.

f) Kupweteka kwambiri kwa muchifuwa kapena kubanika ngakhale kuthamanga kwambiri kwa mtima.

g) Kupweteka kwambiri kwa mutu, chidima kapena kuona mchenga mmaso, kutupa kwa manja ndi nkhope, komanso kupweteka kwambiri kwa pansi pa nthiti kapena kusanza.

h) Kukhumudwa koposa muyeso, kapena kukhala ndi maganizo ozipangira nokha upandu kapena mwana amene mukuyembekezera.

i) Kugunda kwa mwana: Pamene mwayamba kumva kugunda kwa mwana wanu, mukuyenera kumva kugunda kumeneku tsiku lina lililonse. Pakakhala kusintha kwa kagundindwe, pitani kuchipatala chomwe muli nacho pafupi.

Ndikofunikila kuti muzipita ku sikelo mwa ndondomeko pa nthawi yomwe muli ndi pakati. Mukuyenera kukumana ndi namwino kasanu ndi katatu(8). Pa ulendo uli onse, muzaonedwa ndipo muzalangizidwa moyenera malinga ndi zomwe anamwino apeza komanso m'mene mimba yanu yakulira.

Pa nthawi imeneyi, ndikofunikira kukhala opanda nkhawa mmalingaliro anu. Pezani chithandizo mwa nsanga ngati nkhawa yakuvutani polankhula ndi azaumoyo kapena abale ndi alongo owakhulupilira. Nenezani nkhaza ina iliyonse mwachitiridwa chifukwa chokhala mayi.

Onetsetsani kuti mukudya mwa nthanzi, kuphatikizapo kumwa folic acid kuchokera pa nthawi yomwe mukukonzekera kukhala ndi pakati, komanso imwani mankhwala oonjezera magazi ngakhale oonjezera timichele ta nthupi pamene muli ndi pakati.

Gonani mu masikito onyikidwa m'mankhwala komanso landirani mankhwala otetezera ku malungo pamene muli ndi mimba.

Onetsetsani kuti mwalandira katemera yense oyenerera kuchokera ku chipatala.

Pewani kusuta fodya komanso kumwa mowa

Pewani kumwa makhwala aliwonse azisamba, ndipo landilani uphungu oyenela kuchoka kwa azaumoyo a kuchipatala musadamwe mankhwala ena aliwonse.

Mukulimbikitsidwa kubwela ku sikelo limodzi ndi okondedwa anu nthawi zonse
